# The beneficial effects of dietary restriction on learning are distinct from its effects on longevity and mediated by depletion of a neuroinhibitory metabolite

**DOI:** 10.1371/journal.pbio.2002032

**Published:** 2017-08-01

**Authors:** Mihir Vohra, George A. Lemieux, Lin Lin, Kaveh Ashrafi

**Affiliations:** Department of Physiology, University of California San Francisco, San Francisco, California, United States of America; Brandeis University, United States of America

## Abstract

In species ranging from humans to *Caenorhabditis elegans*, dietary restriction (DR) grants numerous benefits, including enhanced learning. The precise mechanisms by which DR engenders benefits on processes related to learning remain poorly understood. As a result, it is unclear whether the learning benefits of DR are due to myriad improvements in mechanisms that collectively confer improved cellular health and extension of organismal lifespan or due to specific neural mechanisms. Using an associative learning paradigm in *C*. *elegans*, we investigated the effects of DR as well as manipulations of insulin, mechanistic target of rapamycin (mTOR), AMP-activated protein kinase (AMPK), and autophagy pathways—processes implicated in longevity—on learning. Despite their effects on a vast number of molecular effectors, we found that the beneficial effects on learning elicited by each of these manipulations are fully dependent on depletion of kynurenic acid (KYNA), a neuroinhibitory metabolite. KYNA depletion then leads, in an N-methyl D-aspartate receptor (NMDAR)-dependent manner, to activation of a specific pair of interneurons with a critical role in learning. Thus, fluctuations in KYNA levels emerge as a previously unidentified molecular mechanism linking longevity and metabolic pathways to neural mechanisms of learning. Importantly, KYNA levels did not alter lifespan in any of the conditions tested. As such, the beneficial effects of DR on learning can be attributed to changes in a nutritionally sensitive metabolite with neuromodulatory activity rather than indirect or secondary consequences of improved health and extended longevity.

## Author summary

Learning capacity is known to decline with age, and similar effects are also associated with several neurodegenerative diseases. Regulation of insulin signaling by dietary restriction (DR) modulates lifespan in many organisms, and it has been also shown to enhance learning and memory. However, the underlying mechanisms of these processes are largely unknown due to the difficulty in disentangling the systemic effects of DR from any potentially brain-specific effects. Here, we have analyzed the molecular effects of dietary restriction in *C*. *elegans* and show that associative learning is enhanced by reducing production of the tryptophan metabolite kynurenic acid (KYNA). KYNA is an antagonist of glutamatergic signaling in neurons, and we find that its depletion in the nervous system upon DR allows for increased activation of an interneuron that is both necessary and sufficient to mediate learning. Furthermore, we show that genetic or pharmacological modulation of diverse pathways known to be involved in the physiological responses to DR also enhance learning by reducing KYNA production, likely via the activation of a specific transcription factor. Finally, we demonstrate that KYNA levels have no effect on organismal lifespan, indicating that the effects of this KYNA-mediated response to dietary restriction is truly specific to brain function and not a secondary consequence of improved health or longevity. As altered KYNA levels are associated with neurodegenerative and psychiatric diseases, our results suggest that this component may be an important modulator of learning and memory in humans as well.

## Introduction

Aging and various neurodegenerative disorders are characterized by progressive declines in learning capacity. There are well-established but poorly understood connections between molecular mechanisms that regulate longevity and those that influence learning and memory [1]. For example, reductions in insulin signaling are associated with lifespan extensions in many species [2,3]. In addition to its metabolic effects, dysregulation of insulin signaling has been implicated in cognitive defects such as Alzheimer disease [4]. In turn, dietary restriction (DR), a dietary intervention that is known to reduce insulin signaling and extend lifespan, is associated with enhancements in learning and memory and delays in cognitive decline, even in the context of neurodegenerative disorders [1,5–7]. However, given that DR or reductions in insulin signaling affect a wide range of cellular and organismal processes that can collectively promote longevity, it is unclear whether the beneficial effects elicited by these manipulations are due to direct effects on mechanisms of learning or due to myriad indirect consequences of lifespan extension and general improvements in neural maintenance and survival [7,8].

*Caenorhabditis elegans* provides an opportunity for investigating the connections between metabolism, aging, and learning. As in mammals, in *C*. *elegans*, various forms of dietary and caloric restriction extend lifespan [2,8]. And many of the findings that helped solidify the beneficial effects of reduced insulin signaling on lifespan extension initially emerged from studies on *C*. *elegans* [2]. Similarly, other manipulations that recapitulate some of the physiological responses to reduced food intake—for example, reductions in certain components of mechanistic target of rapamycin (mTOR) signaling and activation of AMP-activated protein kinase (AMPK), as well as activation of autophagy—promote longevity even when *C*. *elegans* have unlimited access to food [2].

*C*. *elegans* have also been used to investigate molecular underpinnings of learning and memory with paradigms for both short-term and long-term memory [9–12]. Many of the key molecular components implicated in mammalian learning are evolutionarily conserved in *C*. *elegans*, including N-methyl D-aspartate receptors (NMDARs), which are required for spatial memory and long-term potentiation in mice [13] and certain forms of associative learning in *C*. *elegans* [10]. One paradigm for studying short-term, associative learning in *C*. *elegans* is to pair food with butanone, an odorant that in naïve animals is only mildly attractive. This pairing results in subsequent attraction to butanone, which can be scored in a chemotaxis assay [11,12]. The proportion of animals attracted to butanone is calculated as a chemotaxis index, and a learning index is the difference between the chemotaxis index of conditioned animals and that of naïve animals that have not previously been exposed to butanone, which allows for normalized comparisons between treatments or strains that may differ in innate responses to butanone [11,12].

Using the butanone association assay, we investigated the effects of DR and perturbations of molecular mechanisms that change upon restricting nutritional intake—hereafter referred to as DR mimetics—on the learning capacity of *C*. *elegans*. Specifically, we investigated the effects of reductions in either insulin or mTOR signaling pathways, as well as the effects of pharmacological and genetic interventions that lead to activations of AMPK and autophagy. Here, we show that DR and these DR mimetics each result in learning enhancements. Despite their wide-ranging cellular and organismal effects, we find that the beneficial effects of each of these interventions on learning are fully dependent on reductions in kynurenic acid (KYNA), a tryptophan-derived metabolite that can be a competitive antagonist of NMDARs [14]. We identify the neuronal sites of KYNA production and KYNA-responsive, NMDAR-expressing neurons required for learning. Although each of DR, insulin, mTOR, AMPK, and autophagy has been under intensive investigation, their effects on the kynurenine pathway (KP) have remained largely unknown. We show that each of these interventions modulate transcription of a gene encoding key enzyme of the pathway and thereby provide a potential explanation of how molecular mechanisms that function in the periphery of the animal can affect levels of a neuronal metabolite. Finally, we show that changes in KYNA levels do not alter lifespans in the context of any of the dietary, genetic, or pharmacological interventions tested, suggesting that the effects of DR and its mimetics on learning can be disentangled from their broad effects on cellular maintenance and lifespan.

## Results

### NMDAR-dependent activity of a single pair of interneurons promotes learning

**Fig 1:**
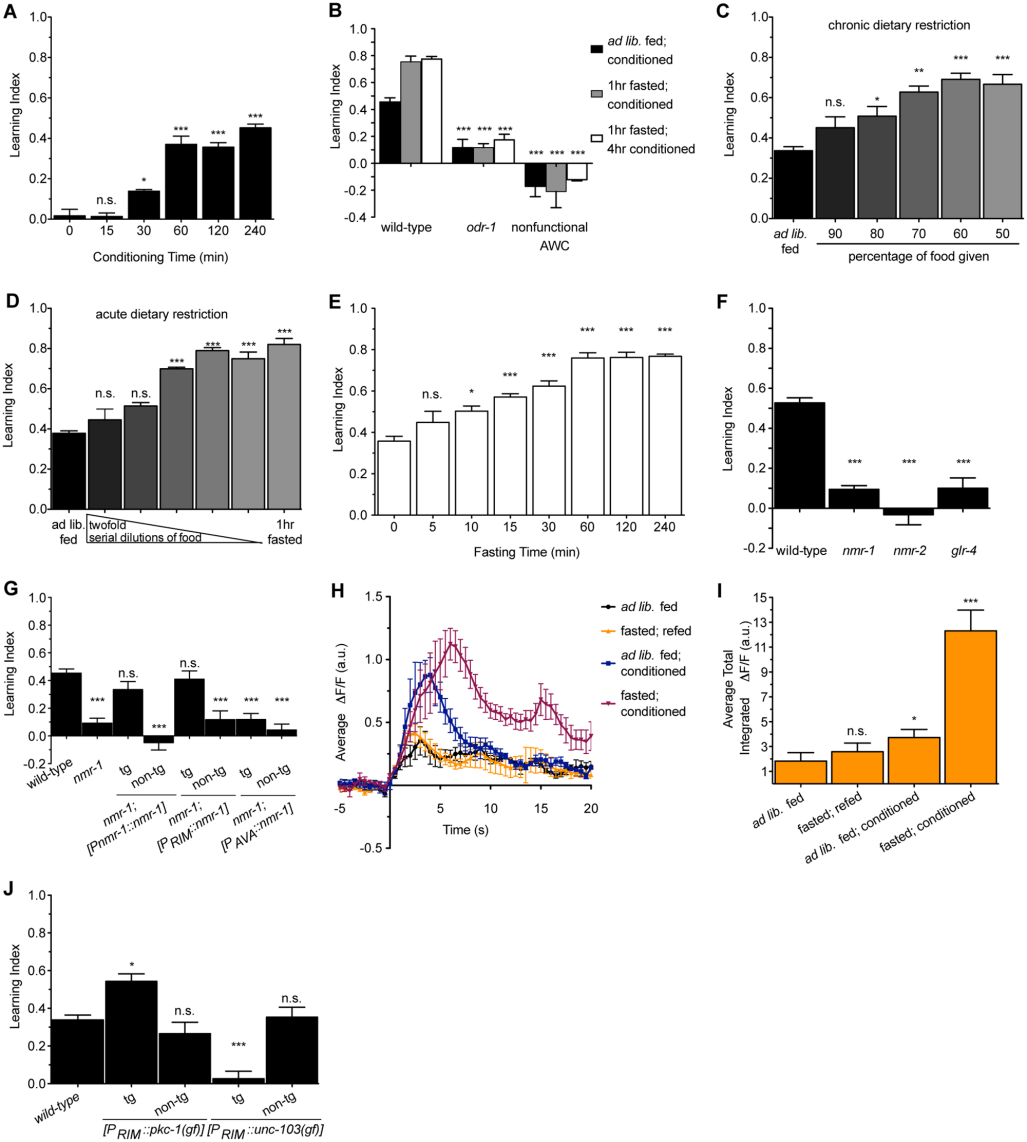
Dietary restriction and activation of RIM interneurons enhance learning. (A) Effects of various durations of conditioning on the learning index of wild-type animals. *n* = 6, *p < 0.05, ***p < 0.001 by 1-way ANOVA (Tukey). (B) Animals mutant in *odr-1* or with nonfunctional AWC neurons fail to learn in response to butanone. *n* = 6, ***p < 0.001 by one-way ANOVA (Tukey). (C) Chronic dietary restriction (DR) (50%–90% of ad libitum food levels for animals’ entire lives) enhance learning. *n* = 3, *p < 0.05, **p < 0.01, ***p < 0.001 by 1-way ANOVA (Tukey). (D) Acute DR (DR for 1 hour) as well as a 1-hour fast enhance learning. *n* = 3–6, ***p < 0.001 by 1-way ANOVA (Tukey). (E) Effects of various durations of fasting prior to a 1-hour conditioning period on learning. *n* = 3–10, *p < 0.05, ***p < 0.001 by 1-way ANOVA (Tukey). (F) Animals mutant in glutamatergic receptors fail to learn. *n* = 6–10, ***p < 0.001 by 1-way ANOVA (Tukey). (G) Reconstitution of *nmr-1* in only the RIM neurons is sufficient to restore learning capacity to *nmr-1* mutants. Learning index values for *nmr-1* mutants with *nmr-1* reconstituted under its own promoter, an RIM-specific promoter, or an AVA-specific promoter are shown. tg denotes transgenic animals; non-tg denotes non-transgenic siblings. *n* = 3, *p < 0.05, **p < 0.01, ***p < 0.001 by 1-way ANOVA (Tukey). (H) Average intensity of spontaneous GCaMP transients in RIM from the 250-second imaging window aligned to a −5-second to 20-second time axis. (I) Average total intensity of RIM GCaMP fluorescence over the entire 250-second imaging window shows that conditioning significantly increases transient intensity, and fasting before conditioning has an even greater effect. *n* = 6–10, *p < 0.05, **p < 0.01, ***p < 0.001 by 1-way ANOVA (Tukey). (J) Chronic activation of RIM by using a constitutively active protein kinase C (PKC) encoded by *pkc-1(gf)* promotes learning even in animals fed ad libitum while silencing of RIM using an overactive potassium channel endcoded by *unc-103(gf)* abolishes learning capacity. tg denotes transgenic animals; non-tg denotes non-transgenic siblings. *n* = 4–9, *p < 0.05, ***p < 0.001 by 1-way ANOVA (Tukey). Animals in panels (F), (G), and (J) were fed ad libitum and conditioned for 1 hour. All data are represented as mean ± SEM. Underlying data can be found in S1 Data.

Associative learning helps animals predict outcomes based on environmental stimuli. We used the well-established butanone learning paradigm to investigate the effects of various manipulations on short-term learning capacity of *C*. *elegans* (S1 Fig) [11,12]. The learned attraction to butanone was notable after 30 min of pairing butanone with the food *Escherichia coli* (conditioning) and reached maximal levels by 1 hour (Fig 1A). This learned attraction was dependent on the previously identified ODR-1 receptor and function of the AWC neurons, the site of ODR-1 function in butanone sensation [15] (Fig 1B).

Associative learning to butanone can be enhanced if animals are given a brief fast before conditioning. This is not simply modulation of the attraction to butanone by fasting, because fasted animals that are re-fed in the absence of butanone do not exhibit any enhanced attraction to butanone [11]. Consistent with these prior results, we found that learned attraction to butanone was progressively enhanced by chronic DR, where animals were grown on decreasing concentrations of food for their entire lives prior to the assay (Fig 1C). The learning-enhancing effects of chronic DR were also observed upon a short-term fast or acute DR, where, immediately before conditioning, animals were cultured with decreasing concentrations of food for 1 hour had a similar effect (Fig 1D). While there was a progressive enhancement of learning by increasing the duration of the fast, additional benefits did not accrue beyond a 1-hour fast (Fig 1E). The extent of the learning enhancement seen with a 1-hour acute DR was the same as that seen in animals that were exposed to chronic DR.

In mammals, members of the ionotropic glutamate receptors of both the N-methyl D-aspartate (NMDA) and the non-NMDA classes are required for learning [13] and, in *C*. *elegans*, a requirement for *glr-1*, encoding a non-NMDA α-amino-3-hydroxy-5-methyl-4-isoxazolepropionic acid (AMPA) receptor (AMPAR), in butanone learning has been previously reported [11]. We found similar requirements for *nmr-1* and *nmr-2*, which encode the 2 subunits of *C*. *elegans* NMDARs [10], and *glr-4*, which encodes a putative kainate receptor homolog [16] (Fig 1F).

While AMPARs and kainate receptors are widely expressed in the *C*. *elegans* nervous system, expression of NMDARs is restricted to only a few neurons, including the RIM and AVA interneurons [17]. Therefore, we chose to focus on the NMDAR-expressing neurons as a strategy for identifying specific neurons that are involved in this learning circuit. We previously found that activity of NMR-1 in AVA but not RIM controls the elevated feeding behavior that animals exhibit post-fast [18]. By contrast, reconstitution of wild-type *nmr-1* in RIM conferred learning ability to *nmr-1* mutants, while reconstitution of *nmr-1* in AVA could rescue post-fast feeding behavior but not learning (Fig 1G and S2A Fig). Thus, while these behaviors reflect NMDAR-dependent food-related plasticity, they are mediated through distinct circuits.

To better understand the function of RIM in learning, we used a genetically encoded calcium reporter, GCaMP3.0, specifically expressed in RIM to measure intracellular Ca^2+^ transients as a proxy for neuronal activity. We first assessed the basal state of RIM activity by measuring spontaneous transients over an extended period (250 s). To compare average intensities of transients, we identified transients during the 250-second imaging window and plotted fluorescence over the following 20 seconds. Similar to other reports [19], spontaneous transients were largely absent in naïve, ad libitum fed animals. However, spontaneous Ca^2+^ transient intensity increased substantially after ad libitum fed animals were conditioned on butanone, and this was even further increased when animals were fasted before conditioning (Fig 1H). We also analyzed Ca^2+^ transients by measuring the total intensity from all transients during the entire 250-second imaging window and found that the results were similar (Fig 1I). In contrast, there was no change in transients when *nmr-1* mutants were similarly fasted and conditioned (S2B and S2C Fig). As it is known that the presence of both NMR-1 and NMR-2 is required for NMDAR function [10,20], *nmr-1* mutants lack functional NMDARs, indicating that learning requires NMDAR activity in RIM. Thus, RIM activity positively correlates with learned attraction to butanone.

To determine a causal relationship between RIM and learning, we genetically controlled RIM activity. We expressed a constitutively active protein kinase C (PKC) to chronically activate RIM [21] and an overactive potassium channel, UNC-103, to chronically silence it [22]. Activating RIM-enhanced butanone learning while silencing RIM blocked it (Fig 1J) without changing naïve chemotaxis (S2D Fig). Thus, not only do RIM’s Ca^2+^ dynamics correlate with learning, but its activity determines the extent to which learning occurs.

### KYNA acts as an NMDAR neuromodulator to regulate learning

**Fig 2:**
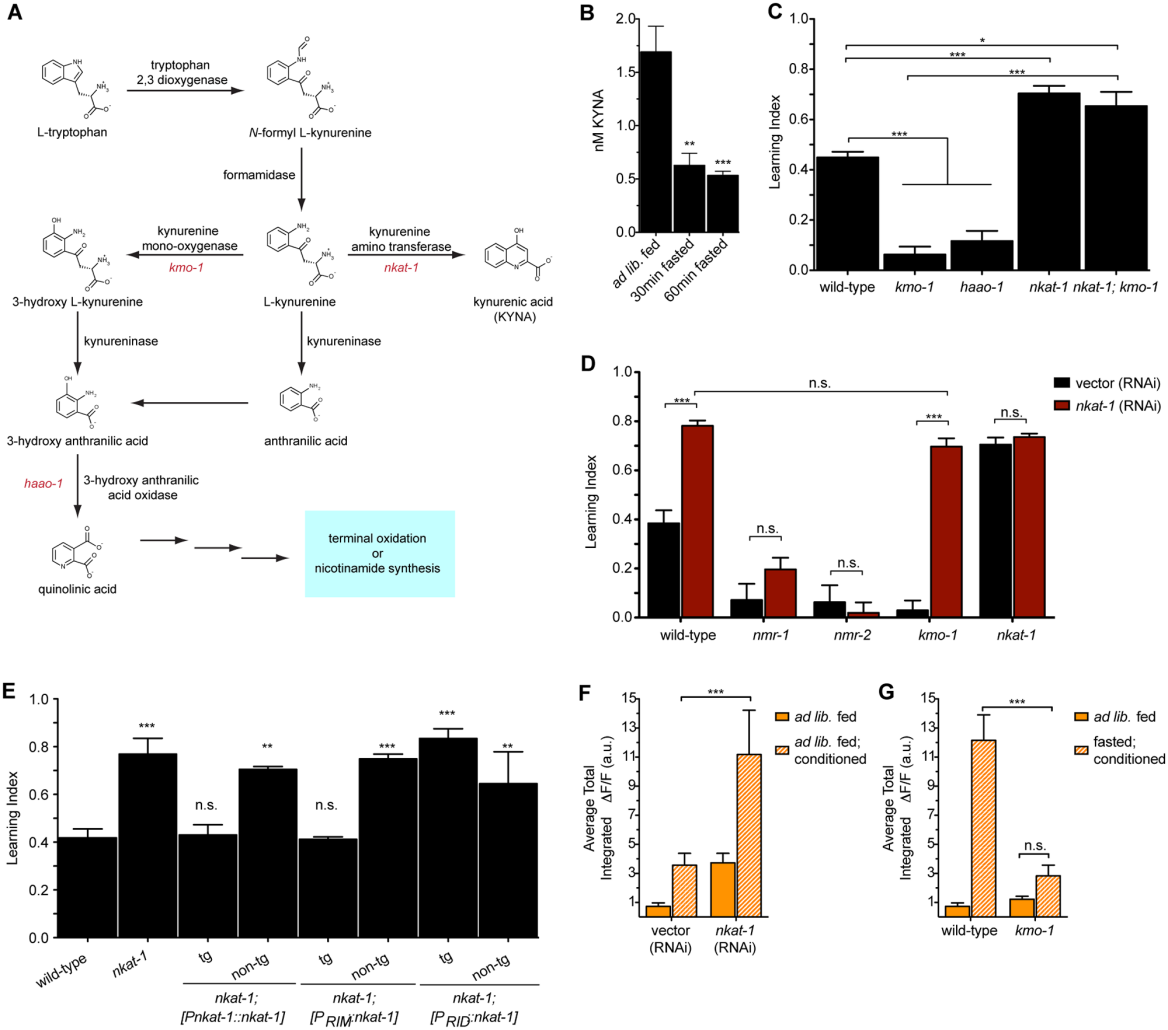
Reduction of neuronally produced kynurenic acid (KYNA) mimics the effects of dietary restriction (DR) on learning. (A) Schematic of the kynurenine pathway (KP). (B) KYNA levels are reduced upon fasting, as indicated by high-performance liquid chromatography (HPLC) measurements of whole-animal extracts. *n* = 5–18, **p < 0.01, ***p < 0.001 by 1-way ANOVA (Tukey). (C) Mutants with reduced KYNA levels have enhanced learning, while those with elevated levels have learning deficits. *n* = 6–10, *p < 0.05, ***p < 0.001 by 1-way ANOVA (Bonferroni). (D) The learning deficits of *kmo-1* mutants but not those of *nmr* mutants are fully reversed by *nkat-1* RNA interference (RNAi). *n* = 3, ***p < 0.001 by 2-way ANOVA (Bonferroni). (E) Learning index values for *nkat-1* mutants with *nkat-1* reconstituted under its own promoter, an RIM-specific promoter, or an RID-specific promoter. tg denotes transgenic animals; non-tg denotes non-transgenic siblings. *n* = 3, *p < 0.05, **p < 0.01, ***p < 0.001 by 1-way ANOVA (Tukey). (F) Average total intensity of RIM GCaMP fluorescence over the entire 250-second imaging window shows that conditioning and exposure to *nkat-1* RNAi results in increased transient intensity. *n* = 6–10, *p < 0.05, **p < 0.01, ***p < 0.001 by 1-way ANOVA (Tukey). (G) Average total intensity of RIM GCaMP fluorescence over the entire 250-second imaging window shows that fasting and conditioning does not increase transient intensity in RIM in *kmo-1* mutants. *n* = 6–10, ***p < 0.001 by 2-way ANOVA (Bonferroni). Animals in panels (C), (D), and (E) were fed ad libitum and conditioned for 1 hour. All data are represented as mean ± SEM. Underlying data can be found in S1 Data.

Given the requirement of NMDARs in the butanone learning paradigm, we next examined KYNA as a potential mechanism by which fasting may promote learning. This seemed possible because the tryptophan-derived KYNA (Fig 2A) is an endogenous NMDAR antagonist [14], and we previously found that, in the context of *C*. *elegans* feeding regulation, KYNA is depleted during fasting, which results in the activation of NMDAR-expressing neurons [18]. To demonstrate that the KYNA levels change in a time frame consistent with the effects of acute fasting on learning enhancement, we extracted KYNA from animals fasted for 30 min or 60 min and found it to be depleted to the same low levels as those reported for 2-hour-fasted animals [18] (Fig 2B). This was consistent with our finding that fasting beyond 1 hour does not further improve learning.

KYNA, a terminal metabolite of the KP, is synthesized from kynurenine by the action of kynurenine amino transferases, encoded by *nkat* genes in *C*. *elegans* [18]. However, if kynurenine is acted upon by kynurenine monooxygenase, encoded by *kmo-1*, and 3-hydroxyanthranilate 3,4-dioxygenase, encoded by *haao-1*, it is converted to the NMDAR agonist quinolinic acid (QA) [23] (Fig 2A). We and others have previously found that inactivation of *nkat-1*, which is expressed in only a small subset of neurons, results in depleted levels of KYNA, while *kmo-1(tm4529)* or *haao-1(tm5627)* mutants have elevated KYNA levels [18,24]. Consistent with a role for KYNA in inhibition of NMDAR signaling, *kmo-1* and *haao-1* mutants had learning defects, while *nkat-1(ok566)* mutants had enhanced learning that was not further elevated by fasting (Fig 2C and S3A Fig). To ascertain whether the learning improvements of *nkat-1* were due to reduced KYNA or elevated QA, we examined *nkat-1; kmo-1* double mutants, which are predicted to be doubly deficient for KYNA and QA biosynthetic capacity. We found them to have enhanced learning, suggesting that an inhibitory role for KYNA predominates in this behavior (Fig 2C).

We considered the possibility that the learning defects of KYNA-replete animals may be due to altered development or damage to learning circuits. However, prolonged conditioning rescued learning defects in *kmo-1* and *haao-1* mutants (S3B Fig). Similarly, fasting, which reduces KYNA levels even in *kmo-1* and *haao-1* mutants, partially reversed their learning deficits (S3A Fig). Chronic DR had similar effects on *kmo-1* mutants (S3C Fig), whereas it did not further elevate *nkat-1* mutants’ learning (S3D Fig). By contrast, neither depleting KYNA (via RNAi interference [RNAi] of *nkat-1*) nor prolonged conditioning conferred a similar increase in learning capacity to the *nmr-1* or *nmr-2* mutants (Fig 2D and S3B Fig). Together, these findings support the notion that NMDARs play a critical role in this learning paradigm and that levels of KYNA modulate the activity of these receptors without being fundamentally required for learning.

In *C*. *elegans*, *nkat-1* and *nmr* genes are expressed in a small subset of neurons that are either in close proximity to each other or, as in the case of RIM, overlapping [18]. To determine the relationship between sites of KYNA production and the NMDAR-dependent response of RIM neurons to this neuromodulatory metabolite, we reconstituted *nkat-1* in subsets of neurons in which *nkat-1* is normally expressed. Reconstitution of *nkat-1* in RIM was sufficient to blunt the enhanced learning of *nkat-1* mutants. In contrast, reconstitution in another pair of *nkat-1*-expressing neurons, RID, had no effect on learning but could rescue *nkat-1* mutants’ hyperactivated feeding (Fig 2E and S3E Fig). Given that KYNA has been shown to directly bind to the glycine site of NMDARs to decrease their activity [14], our findings are consistent with KYNA regulation of NMDAR activity in an autocrine fashion but do not rule out more complicated possibilities, including indirect effects on NMDAR activity (for example, through modulation of receptors) that, in turn, affect the release of glutamate or other agonists of NMDARs.

Finally, we found that KP metabolites affect Ca^2+^ influx into RIM in a manner consistent with behavioral results. As before, naïve, ad libitum fed animals on vector RNAi exhibited very little Ca^2+^ influx, which was increased upon conditioning (Fig 2F). Depletion of KYNA via *nkat-1* RNAi resulted in animals with already elevated Ca^2+^ transients that were further increased upon conditioning (Fig 2F and S3F Fig). The elevated Ca^2+^ transients of ad libitum fed, KYNA-deficient animals were similar to those seen in wild-type animals that were fasted and conditioned (Fig 2G). By contrast, elevation of KYNA via *kmo-1* mutation largely abrogated the elevation of Ca^2+^ transients seen upon conditioning of fasting animals (Fig 2G and S3G Fig). Together with previous results, these data are consistent with a model whereby levels of KYNA modulate responsiveness of RIM neurons in an NMDAR-dependent fashion.

### KYNA also modulates NMDAR-dependent aversive learning behaviors

**Fig 3:**
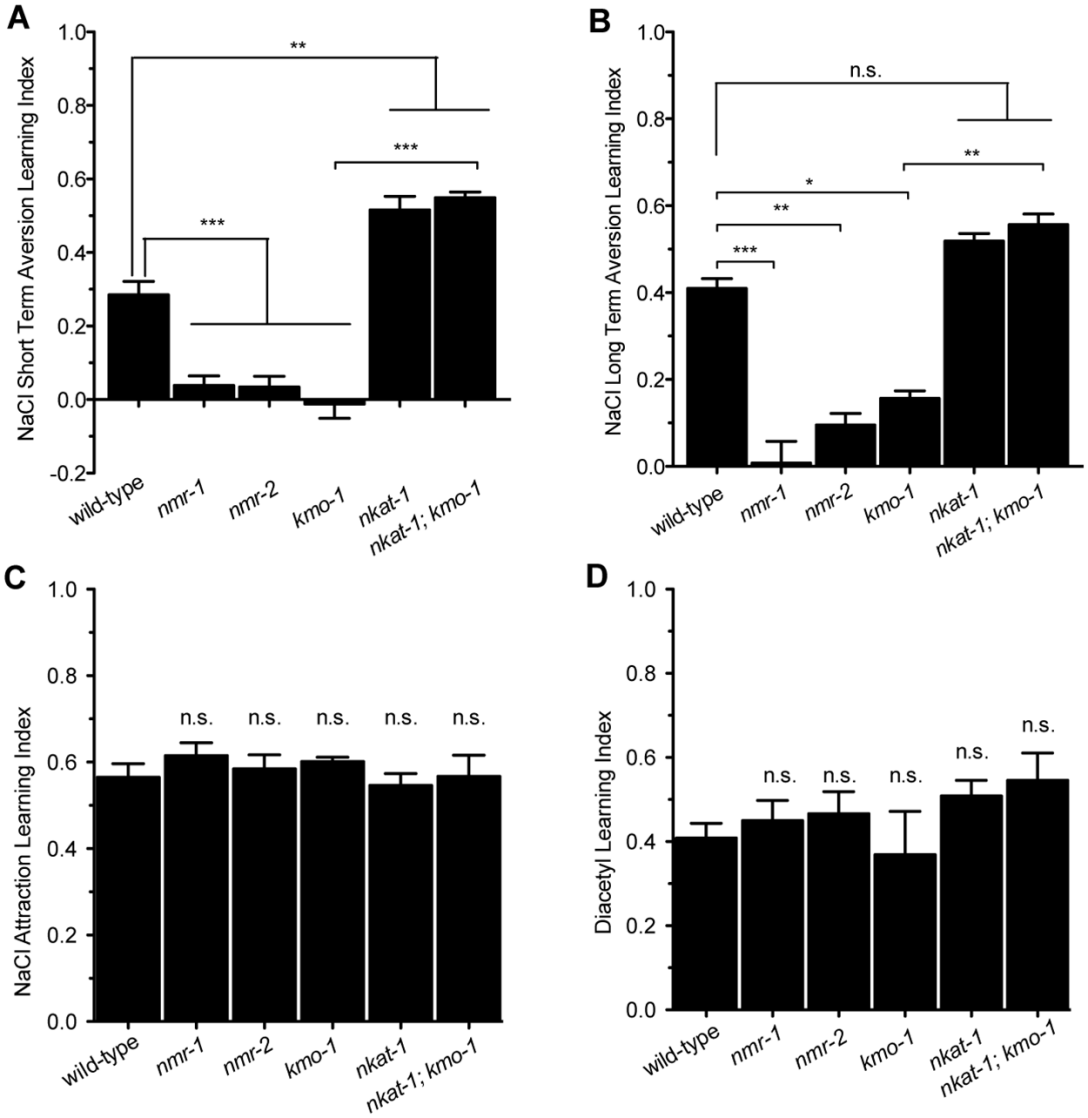
Kynurenic acid (KYNA) depletion enhances learning only in paradigms that require N-methyl D-aspartate receptors (NMDARs). (A) NaCl aversion short-term training: learning index values for animals conditioned with high NaCl without food for 3 hours. *n* = 3–6, *p < 0.05, ***p < 0.001 by 1-way ANOVA (Bonferroni). (B) NaCl aversion long-term training: learning index values for animals conditioned with high NaCl without food for 6 hours. *n* = 3–6, *p < 0.05, ***p < 0.001 by 1-way ANOVA (Bonferroni). (C) NaCl attraction short-term training: learning index values for animals conditioned with high NaCl with food for 6 hours. *n* = 3–6, significance measured by 1-way ANOVA (Tukey). (D) Diacetyl short-term training: learning index values for animals conditioned with the odor diacetyl with food. *n* = 3, significance measured by 1-way ANOVA (Tukey). All data are represented as mean ± SEM. Underlying data can be found in S1 Data. n.s., not significant.

*nmr-1* and *nmr-2* are known to mediate aversive learning when *C*. *elegans* are exposed to high NaCl concentrations without food [10]. As with attractive butanone learning, the ability of animals to learn the aversive signal of high NaCl was diminished with excess levels of KYNA but promoted upon KYNA depletion (Fig 3A). Moreover, extending the conditioning period could enhance learning of wild-type animals to the level of *nkat-1* mutants but had no effect on *nmr-1* or *nmr-2* mutants (Fig 3B). Fasting animals in a normal NaCl environment to deplete KYNA and subsequently conditioning them with high NaCl also enhanced learning in wild-type and *kmo-1* animals to the level of *nkat-1* mutants with no effect on *nmr-1* or *nmr-2* mutants (S4 Fig). Thus, KYNA-mediated modulation of learning is independent of the sensory modality or valence of the paradigm: elevated KYNA dampens learning and reduced KYNA enhances learning, regardless of whether the stimulus is olfactory or gustatory and whether the learned association is attractive or aversive.

Further supporting an NMDAR-dependent mechanism for effects of KYNA on learning, we found that altering KYNA or QA levels had no effect on learning in 2 paradigms in which NMDAR activity is not required: learned attraction when food was paired with either high NaCl concentrations or the odor diacetyl [25,26] (Fig 3C and 3D). Thus, KYNA-mediated modulation of learning is a general phenomenon that occurs in NMDAR-dependent paradigms.

### Genetic and pharmacological manipulations that mimic aspects of DR enhance learning

**Fig 4:**
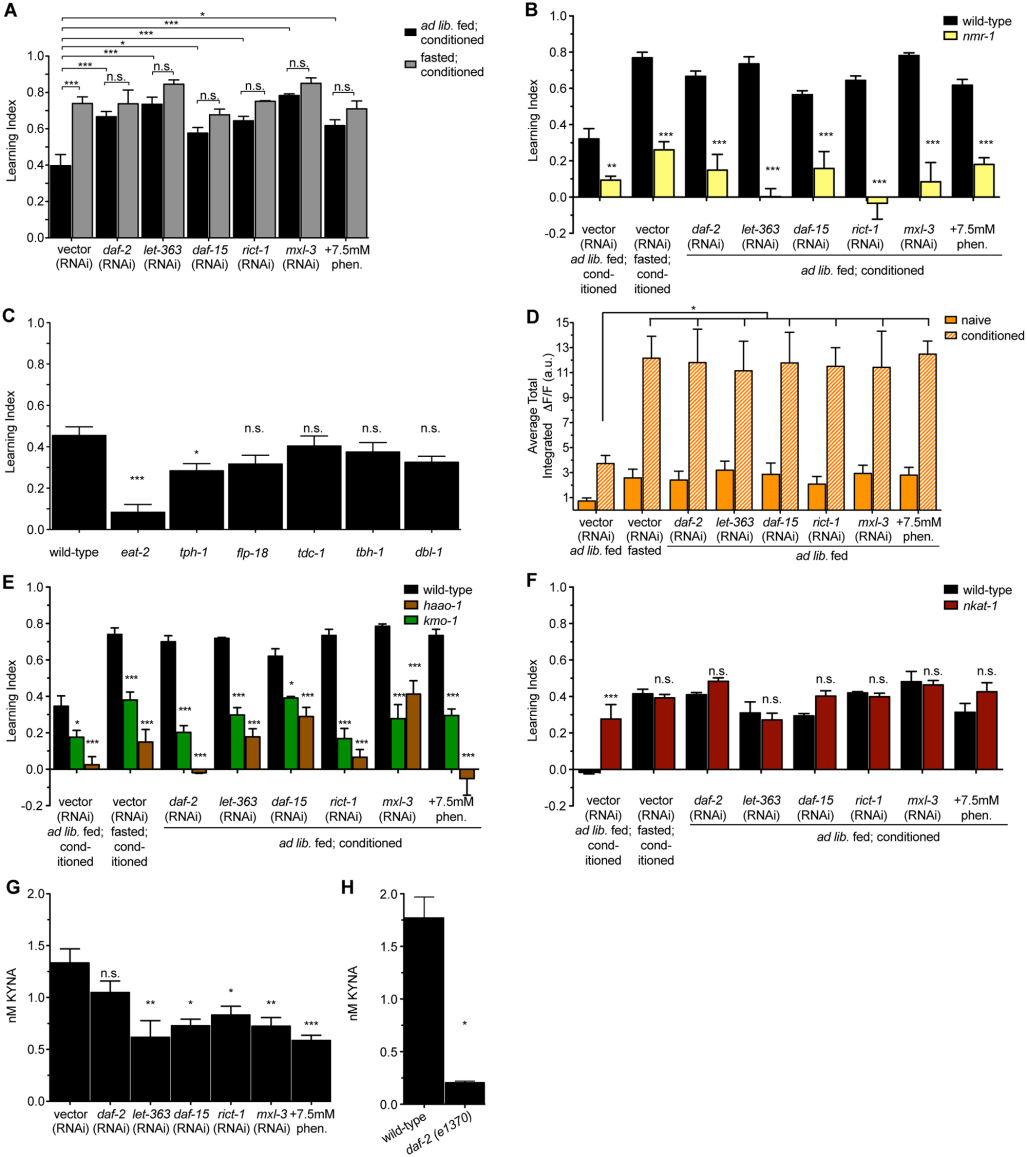
Genetic and pharmacological manipulations that mimic dietary restriction (DR) enhance learning by depleting kynurenic acid (KYNA). (A) RNAi interference (RNAi)-mediated reductions in the insulin receptor (*daf-2*), the mechanistic target of rapamycin (mTOR) kinase (*let-363*), Raptor (*daf-15*), Rictor (*rict-1*), and a negative regulator of autophagy (*mx1-3*), as well as animals treated with an activator of AMP-activated protein kinase (AMPK) (phenformin), have enhanced learning capacity even when fed ad libitum. *n* = 3–6, *p < 0.05, ***p < 0.001 by 2-way ANOVA (Bonferroni). (B) The elevated learning capacities of genetic and pharmacological mimetics of DR are dependent on N-methyl D-aspartate receptor (NMDAR) signaling. *n* = 3, *p < 0.05, **p < 0.01, ***p < 0.001 by 2-way ANOVA (Bonferroni). (C) Learning index values for additional mutants in various neural nutrient sensing pathways: *eat-2* mutants have a pharyngeal pumping defect, *tph-1* mutants do not produce serotonin, *flp-18* mutants lack a neuropeptide Y-like peptide, *tdc-1* mutants do not produce tyramine or octopamine, *tbh-1* mutants do not produce octopamine, and *dbl-1* mutants lack a transforming growth factor β (TGFβ) ligand. *n* = 3–6, *p < 0.05, ***p < 0.001 by 1-way ANOVA (Tukey). (D) Average total intensity of RIM GCaMP fluorescence over the entire 250-second imaging window in animals exposed to genetic and pharmacological DR mimetics. *n* = 6–10, *p < 0.05, **p < 0.01, ***p < 0.001 by 1-way ANOVA (Tukey). (E) Learning index values for mutants with high KYNA exposed to genetic and pharmacological DR mimetics. *n* = 3, *p < 0.05, **p < 0.01, ***p < 0.001 by 2-way ANOVA (Bonferroni). (F) Learning index values for wild-type and *nkat-1* animals given DR mimetics. To ensure that effects of DR mimetics in the context of KYNA depletion could be observed, animals were conditioned for only 15 minutes. *n* = 3, ***p < 0.001 by 2-way ANOVA (Bonferroni). (G) High-performance liquid chromatography (HPLC) measurements of steady-state KYNA levels for animals exposed to genetic and pharmacological DR mimetics. *n* = 5–18, *p < 0.05, **p < 0.01, ***p < 0.001 by 1-way ANOVA (Tukey). (H) HPLC measurements of steady-state KYNA levels for wild-type and *daf-2(e1370)* mutant animals. *n* = 2, *p < 0.05 by 2-tailed Student *t* test. Animals in panels (B), (C), (E), and (F) were ad libitum fed and conditioned. All data are represented as mean ± SEM. Underlying data can be found in S1 Data. n.s., not significant.

While many molecular components underlying learning are known and many mutations that impair learning have been studied, relatively few genetic manipulations are known to improve learning. One such manipulation is impairment of insulin signaling [11,27]. Consistent with this, we found down-regulation of the *C*. *elegans* insulin receptor *daf-2* via RNAi was associated with improved learning in ad libitum fed animals with no further improvements upon fasting (Fig 4A). We reasoned that manipulating other nutritionally sensitive pathways might similarly identify molecular manipulations that improve learning. The mTOR1 and mTOR2 complexes sense nutrient availability and regulate processes ranging from fat storage and protein synthesis to development and lifespan [28]. We found that RNAi-mediated inactivations of *let-363* (encoding the *C*. *elegans* mTOR homolog), *daf-15* (encoding raptor), and *rict-1* (encoding rictor) each led to elevated levels of learning in ad libitum fed animals (Fig 4A). This finding was reminiscent of mammalian studies reporting that chronic mTOR inhibition has procognitive effects [29,30]. Similarly, we found improved learning upon inactivation of the transcription factor *mxl-3*, which leads to autophagy and lipolysis in intestinal cells in response to nutrient deprivation [31], or upon treatment with phenformin, a biguanide compound that activates AMPK, a master regulator of energy homeostasis [32] (Fig 4A). None of these enhanced learning capacities were further improved by fasting (Fig 4A). All of these learning enhancements, however, required *nmr-1* (Fig 4B).

Enhanced learning capacity was not a general feature of manipulating food-related signaling pathways, as mutations in various biogenic amine or peptidergic signaling pathways implicated in other food-related plasticity behaviors had minor or no effects on learning. These included mutations in genes required for synthesis of serotonin (*tph-1*), that of a neuropeptide Y-like molecule (*flp-18*), octopamine (*tbh-1*), both tyramine and octopamine (*tdc-1*), and synthesis of a transforming growth factor β (TGFβ) superfamily member ligand (*dbl-1*). Each of these pathways have been implicated in various food-related behaviors: for example, reductions in serotonin are associated with reduced food levels or increased population density [33,34]. Similarly, tyramine, octopamine, and neuropepetide Y-like signaling pathways are thought to be active when *C*. *elegans* are food deprived [18,35,36]. Moreover, while DR or changes in insulin, mTOR, AMPK, and autophagy can extend lifespan, not all long-lived mutants had enhanced learning. Interestingly, *eat-2* mutants, which are frequently used as a model of DR because of their defects in food intake and prolonged lifespan [37–39], failed to learn (Fig 4C). The reason for this failure is not known; however, given that *eat-2* mutants have defects in cholinergic signaling [37], it is possible that cholinergic signaling plays roles in the development or function of the learning circuit in addition to its role in promoting pharyngeal neuromuscular contractions required for feeding. Of note, *eat-2* mutants were previously shown to have defective long-term memory but normal learning capacity [11]. The reason for the discrepancy between our results and the previous publication is not known, although both studies indicate that mutations in *eat-2* do not confer enhancements on short-term learning.

### The elevated learning capabilities of DR mimetics are KYNA dependent

We next sought to better understand the relationship of KYNA to learning enhancements caused by DR mimetics. As in fasting or depletion of KYNA, the learning enhancements seen in DR mimetics even in the presence of plentiful food supplies correlated with increases in Ca^2+^ transient intensity in RIM similar to control animals that had been fasted before conditioning (Fig 4D and S4 Fig). In each case, elevation of KYNA levels using either *haao-1* or *kmo-1* mutants caused a significant reduction in learning capacity (Fig 4E). We next exposed *nkat-1* mutants to various DR mimetic treatments. However, given that *nkat-1* mutants already have elevated learning, we decreased the time animals were conditioned with butanone to avoid being confounded by a ceiling in our ability to measure learning. Under these conditions, wild-type animals did not learn, but fasted or KYNA-deficient animals still did, albeit to a lesser degree than when the conditioning was for the standard 1-hour period used elsewhere in this study (Fig 4F). Treatment of *nkat-1* mutants with fasting or exposure to any of the DR mimetics did not lead to further improvements in learning (Fig 4F), suggesting that DR mimetics and *nkat-1* mutants function in the same pathway to enhance learning.

We next employed direct biochemical measurements of KYNA levels extracted from populations of whole animals exposed to each of the DR mimetics. As in the case of fasted animals, we found that with each of the DR mimetics, KYNA levels were already depleted, even under ad libitum fed conditions (Fig 4G). While there was a trend of decreased KYNA levels in animals exposed to *daf-2* RNAi, the reduction was not statistically significant. Since these metabolite measurements were conducted on extracts from populations of animals and we could not be certain of the efficacy of *daf-2* RNAi throughout the population, we used *daf-2(e1370)* mutants. KYNA levels of ad libitum fed *daf-2* mutants were substantially reduced compared to those of wild-type animals (Fig 4H).

### DR mimetics alter expression levels of KP genes

Although each of insulin, mTOR, AMPK, and autophagy are extensively studied, mechanisms through which these manipulations could result in changes in the KP have not been established. Moreover, while molecular components of insulin, mTOR, and AMPK signaling pathways are broadly expressed in *C*. *elegans*, the transcriptional regulator MXL-3 acts in the intestine [31], yet its inactivation leads to reduced KYNA levels and enhanced learning. Thus, there must be mechanisms that regulate KYNA levels even when working at sites distant from the neurons involved in KYNA production.

**Fig 5:**
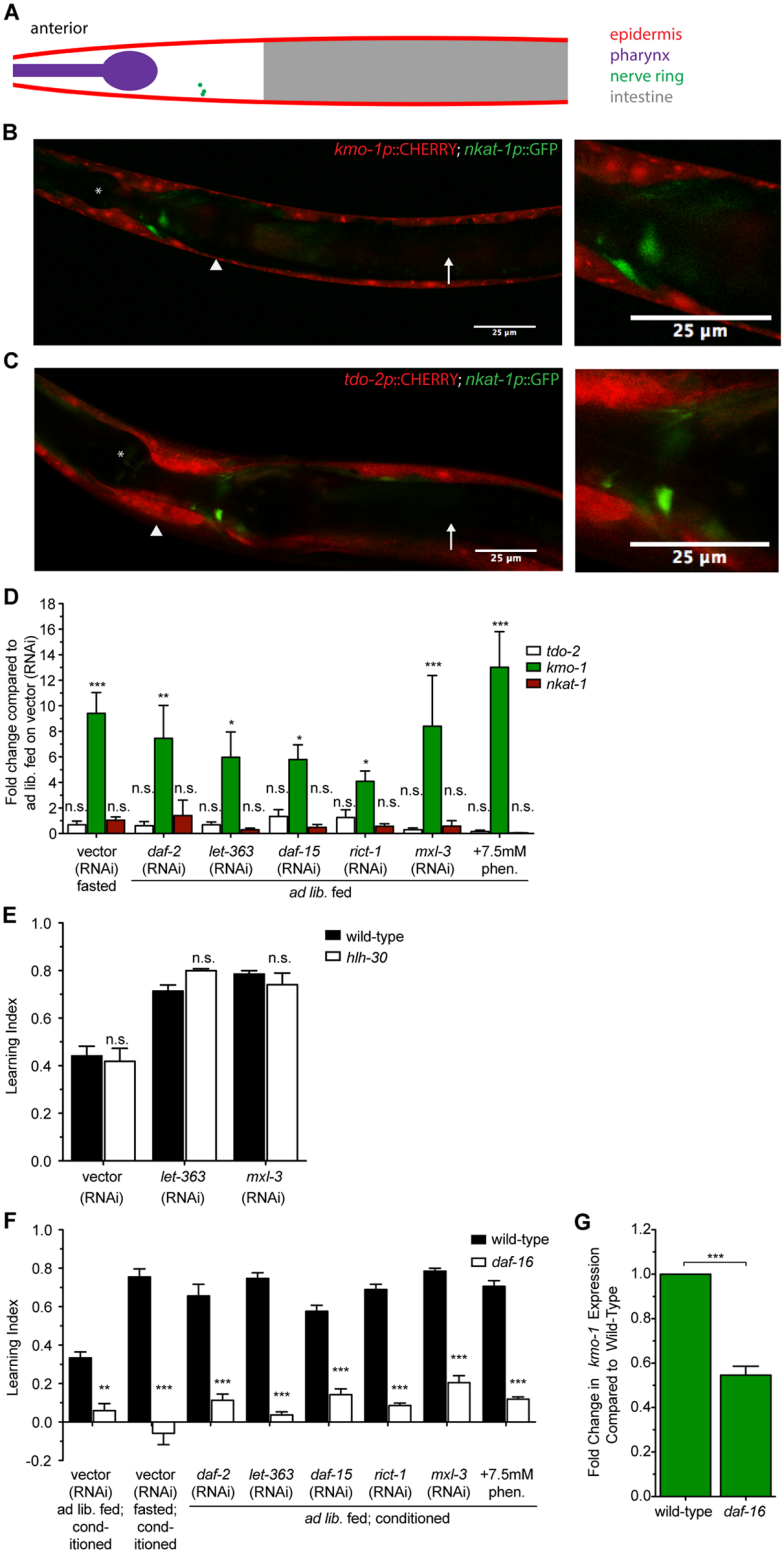
*kmo-1* and *nkat-1* have distinct tissue expression patterns and exhibit distinct transcriptional patterns of regulation. (A) Cartoon representation of the anterior portion of adult *C*. *elegans* comparable to images in panels (B) and (C). Labeled anatomical structures correspond to those in panels (B) and (C). (B) Five-μm thick z-projection of an adult animal with mCherry under control of the *kmo-1* promoter and green fluorescent protein (GFP) under control of the *nkat-1* promoter. Left: anterior portion of the animal. Right: magnified view of the nerve ring showing no overlap of GFP and mCherry. Asterisk indicates pharynx; arrowhead indicates epidermis; arrow indicates intestine. (C) 5-μm thick z-projection of an adult animal with mCherry under control of the *tdo-2* promoter and GFP under control of the *nkat-1* promoter. Left: anterior portion of the animal. Right: magnified view of the nerve ring showing no overlap of GFP and mCherry. Asterisk indicates pharynx; arrowhead indicates epidermis; arrow indicates intestine. (D) Change in transcript levels of kynurenine pathway (KP) genes in animals treated with dietary restriction (DR) or DR mimetics as determined by real-time quantitative PCR (qPCR). Data are represented as fold change compared to ad libitum fed animals on vector (RNA interference [RNAi]). *n* = 3 biological replicates, *p < 0.05, **p < 0.01, ***p < 0.001 by 1-way ANOVA (Tukey). (E) Learning index values for *hlh-30* mutants on *let-363* and *mxl-3* RNAi. *n* = 3, significance measured by 2-way ANOVA (Bonfferoni). (F) Learning index values for *daf-16* mutants on DR mimetics. *n* = 3, **p < 0.01, ***p < 0.001 measured by 2-way ANOVA (Bonferroni). (G) Change in *kmo-1* transcript levels in *daf-16* mutants as determined by real-time qPCR. Data are represented as fold change compared to wild-type. *n* = 3 biological replicates, *p < 0.05, ***p < 0.001 by 1-way ANOVA (Tukey). All data are represented as mean ± SEM. Underlying data can be found in S1 Data.

To better understand mechanisms that regulate flux through the KP, we first considered sites of expression of key enzymes that are required for generation or utilization of kynurenine, the substrate from which KYNA is produced. It has been previously reported that the *tdo-2* gene encoding the first enzyme in the KP is expressed in the body wall muscle and skin-like epidermis of *C*. *elegans* [24]. Indeed, using a transcriptional fusion of the *tdo-2* promoter to green fluorescent protein (GFP), we observed robust expression in the epidermis (Fig 5A and 5C). We found that *kmo-1* shows epidermal expression as well (Fig 5B). Neither *tdo-2* nor *kmo-1* appeared to be expressed in the neurons that express *nkat-1* (Fig 5B and 5C). This suggests that kynurenine must be transported across issues in order for RIM to produce KYNA.

We next examined *tdo-2*, *kmo-1*, and *nkat-1* transcripts via quantitative PCR (qPCR) in animals given DR mimetics. Compared to ad libitum fed animals on vector RNAi, fasting and DR mimetics caused no significant changes in either *tdo-2* or *nkat-1* expression. In contrast, each of the mimetics resulted in a significant increase in *kmo-1* expression (Fig 5D). Given coincident tissue expressions of *tdo-2* and *kmo-1* in a large tissue such as the epidermis, these data indicate that up-regulation of *kmo-1* could compete with KYNA production by shunting kynurenine, the common substrate between NKAT-1 and KMO-1, down a different branch of the KP, resulting in reduced KYNA levels (Fig 2A).

To further understand the relationship between *kmo-1* expression and DR, we set out to investigate the effects of several transcription factors with key roles in DR on *kmo-1* expression. It is well established that insulin signaling causes functional inactivation the FOXO transcription factor DAF-16 and that many of the consequences of reduced insulin signaling seen in *daf-2* mutants require *daf-16* [3]. Similarly, mTOR and AMPK signals can be transduced via the NRF2 master regulator SKN-1 [3]. Additionally, mTOR activity affects HLH-30, a TFEB orthologue that competes with MXL-3 for binding sites and exerts opposing effects, and fasting increases *hlh-30* expression [31,40]. Finally, cross-talk among many of these pathways has been shown. Thus, we sought to determine the effects of *daf-16*, *skn-1*, and *hlh-30* reduction-of-function mutations on learning. We found that *skn-1* mutants failed to chemotax, so they could not be properly assayed. Although other phenotypes resulting from loss of *let-363* and *mxl-3* are known to involve *hlh-30* [31], *hlh-30* mutants had no learning phenotype (Fig 5E), suggesting it may not play a role in regulating KYNA production relevant for this behavior. In contrast, *daf-16* mutants had a learning defect that blocked the enhancements of fasting (Fig 5F). Moreover, loss of *daf-16* not only blocked the enhanced learning of insulin-deficient animals, it abrogated the enhanced learning of various DR mimetics (Fig 5F), raising the possibility that insulin signaling pathway serves as a major link between various nutritionally sensitive pathways and the KP. Consistent with this, we found that, compared to wild-type, *daf-16* mutants had significantly reduced *kmo-1* transcript levels (Fig 5G). It is currently unknown whether *kmo-1* is a direct target of DAF-16.

### Effects of KYNA on learning are independent of lifespan effects

We considered the possibility that the effects of KYNA levels on learning may be secondary consequences of overall improvement of animal health and lifespan. Despite dramatically increased and reduced levels of KYNA, respectively, *kmo-1* and *nkat-1* mutants had wild-type lifespans. Furthermore, while reduction in insulin signaling, inactivation of various components of mTOR, activation of autophagy, or treatment of phenformin had a variety of effects on lifespan, none of their lifespans were altered by *kmo-1* or *nkat-1* mutations (Table 1 and S5 and S6 Figs). Our results indicate that the beneficial effects of DR on learning in *C*. *elegans* can be accounted for by reductions in KYNA and that this is independent of the effects of DR or its mimetics on lifespan.

**Table 1:** Median lifes pans of animals on DR mimetics. Median lifespans of animals given dietary restriction (DR) mimetics are not affected by *kmo-1* or *nkat-1* mutations. Significance was measured by logrank tests. See also S5 and S6 Figs for full survival curves.

RNAi	Strain	Replicate 1 (S6 Fig)		Replicate 2 (S7 Fig)	
		*n*	Median lifespan	*n*	Median life span
Vector	wild-type	86	17	58	17
	*kmo-1*	88	17 (n.s.)	60	19 (n.s.)
	*nkat-1*	73	17 (n.s.)	58	17 (n.s.)
*daf-2*	wild-type	83	27	60	29
	*kmo-1*	75	29 (n.s.)	59	29 (n.s.)
	*nkat-1*	77	27 (n.s.)	60	29 (n.s.)
*let-363*	wild-type	81	22	60	24
	*kmo-1*	76	24 (n.s.)	58	24 (n.s.)
	*nkat-1*	78	22 (n.s.)	62	24 (n.s.)
*daf-15*	wild-type	75	20	60	24
	*kmo-1*	78	20 (n.s.)	60	24 (n.s.)
	*nkat-1*	81	20 (n.s.)	60	24 (n.s.)
*rict-1*	wild-type	83	13	61	15
	*kmo-1*	79	13 (n.s.)	57	15 (n.s.)
	*nkat-1*	82	13 (n.s.)	60	15 (n.s.)
*mxl-3*	wild-type	75	20	77	20
	*kmo-1*	80	20 (n.s.)	74	20 (n.s.)
	*nkat-1*	72	17 (n.s.)	69	20 (n.s.)
vector + 7.5-mM phen.	Wild-type	76	22	60	24
	*kmo-1*	76	20 (n.s.)	60	26 (n.s.)
	*nkat-1*	79	22 (n.s.)	68	24 (n.s.)

**Abbreviations:** n.s., not significant; RNAi, RNA interference

## Discussion

We have identified several pathways that enhance learning in *C*. *elegans*. In addition to previously reported effects of impaired insulin signaling, we found that decreased mTOR signaling, activated intestinal autophagy, and AMPK activation each lead to learning enhancements in well-fed animals that are similar in magnitude to the levels seen when animals are exposed to DR or a short fast. Because changes in insulin, mTOR, autophagy, and AMPK are all features of the physiological changes induced by DR, we termed them DR mimetics and predicted that they might share common mechanisms of modulating learning. This was indeed the case, as all of these DR mimetics enhanced learning via depletion of KYNA in the nervous system and consequent activity of a specific NMDAR-expressing neuron, RIM. Simply depleting KYNA mimics the effects of DR on learning, while KYNA elevation completely abrogates the learning enhancements caused by DR mimetics. Importantly, while the changes in KYNA were necessary and sufficient to account for the improvements in learning, KYNA levels did not alter lifespan in any of the conditions tested. Thus, the effects of DR on learning can be ascribed to a very specific mechanism rather than myriad benefits associated with DR.

Unlike the NMDARs, which were absolutely required for learning in this model, KYNA levels modulated the speed at which learning occurred, rather than being critically required for the occurrence of learning at all. KYNA depletion did not result in naïve attraction to butanone or aversion to NaCl but accelerated the rate at which the associations were formed. This is supported by the observation that *nkat-1* RNAi and DR mimetics that depleted KYNA could still elicit learning even with a shortened conditioning period that was insufficient for learning in wild-type animals (Fig 4F). Furthermore, the learning deficits of *kmo-1* or *haao-1* mutants with high KYNA could be overcome with a longer conditioning period (S3B Fig).

**Fig 6:**
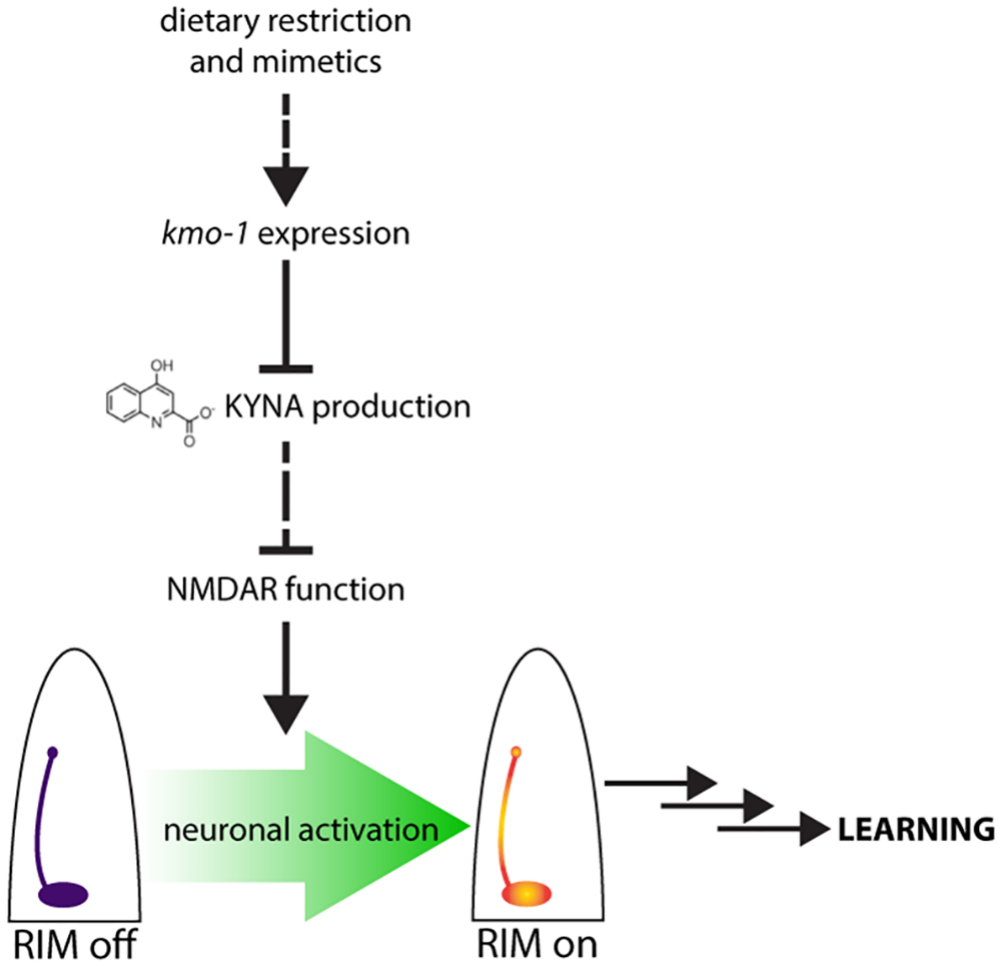
Model for how dietary restriction (DR) and DR mimetics enhance learning. DR and DR mimetics decrease production of kynurenic acid (KYNA). This acts as a release of inhibition on N-methyl D-aspartate receptor (NMDAR) function. The result is a greater influx of Ca^2+^ into the RIM interneurons after conditioning, ultimately leading to learning.

Our data are consistent with a model whereby signals that promote activity of NMDARs in RIM neurons compete with locally produced KYNA, which antagonizes these receptors. This signaling balance determines the timeframe in which learning occurs. Genetic, pharmacological, or nutritional manipulations that enhanced learning did so by depleting the antagonizing effects of KYNA on NMDAR signaling (Fig 6).

The precise mechanisms by which KYNA antagonizes NMDARs remains unsettled. Two competing yet not mutually exclusive mechanisms for antagonistic effects on glutamatergic signaling have been proposed. One is based on the ability of KYNA to directly bind to the glycine-binding site of NMDARs [14], albeit with low affinity, making KYNA the only known potential endogenous antagonist of NMDARs. Another proposed mechanism is that KYNA reduces glutamate release via antagonism of α7 nicotinic acetylcholine receptors (α7nAChRs) [41,42]. While the combination of genetic, direct metabolite measurement, and neuronal imaging studies strongly indicated that KYNA exerts an inhibitory effect on NMDARs, our data cannot distinguish between direct and indirect effects. However, the finding that RIM is both the site of production of KYNA and KYNA-responsive NMDARs is consistent with KYNA being a direct, low-affinity, noncompetitive antagonist of NMDARs.

We found that KYNA depletion enhanced both attractive and aversive learning behaviors. Moreover, we found that reconstitution of *nmr-1* in RIM was sufficient to confer learning to *nmr-1* mutants in the butanone learning paradigm, and it has been reported that *nmr-1* activity in RIM is required for aversive NaCl learning [10]. Butanone is sensed by the AWC neurons, while NaCl is sensed by ASE neurons. The fact that NMDAR function in RIM directs both attractive and aversive learning suggests that RIM activity generally enhances the ability of the *C*. *elegans* nervous system to form associations in response to environmental stimuli, even when those stimuli are sensed by distinct sensory neurons. Furthermore, we demonstrated the requirement of RIM in learning by artificially silencing it while artificial activation of RIM in the presence of butanone resulted in enhanced learning but did not confer any naïve attraction to this odor. Thus, activity of RIM promotes learning, but environmental cues dictate what the learned behavior is.

By modulating activity of RIM-localized NMDARs, KYNA levels provide a general strategy for linking metabolic state to learning capacity. Despite the highly localized requirement for KYNA in learning, its production could be affected by factors that act in tissues distant from the neuronal sites of KYNA production. Our findings raise the possibility that substrate availability can serve as a mechanism for linking peripherally acting pathways to neuronal KYNA production. In support of this, we found that *tdo-2* and *kmo-1*, encoding for enzymes that are required for generation and utilization of kynurenine, respectively, have coincident expression patterns in the *C*. *elegans* epidermis, a relatively large tissue. In contrast, we found no evidence of expression of these genes in the few neurons that express *nkat-1*. Surprisingly, while each of DR, insulin, mTOR, AMPK, and autophagy has been intensely investigated, their effects on flux through the KP have remained largely unexplored. DR, as well as each of the DR mimetics that enhance learning, caused a significant up-regulation of *kmo-1*, potentially through the transcription factor FOXO/DAF-16. Since KMO-1 and NKAT-1 both utilize kynurenine as a substrate, the anatomical site of function of *kmo-1*, as well as its up-regulation, deprive NKAT-1 of access to kynurenine to produce KYNA. Of course, additional layers of regulation, including those that directly impinge on the activity of NKAT-1 and those that function in transport of metabolic intermediates across tissues, are also potential mechanisms by which distally acting factors could regulate KYNA levels. Reminiscent of the *C*. *elegans* findings, data in mammalian systems also indicates that manipulation of the KP in peripheral tissues can affect brain levels of the metabolites [43].

There is compelling evidence that, in mammals as in *C*. *elegans*, the KP has significant effects on mechanisms of neural plasticity and learning [44]. Mice lacking the primary KYNA-producing enzyme in the brain have enhanced hippocampal-dependent learning; hippocampal slices from these animals have increased extracellular glutamate and exhibit greater long-term potentiation amplitude in an NMDAR-dependent manner [45]. In contrast, adding exogenous KYNA to the brain impairs spatial learning and lowers extracellular glutamate levels in rats [46]. Additionally, dysregulation of the KP in humans has been implicated in Alzheimer disease [47,48], Huntington disease [49,50], Parkinson disease [51], depression [52], and schizophrenia [53]. Since changes in KP metabolite levels can slow progression of neurodegeneration in experimental animal systems, a potential direct link between the KP and diverse neurological disorders has been suggested [24,54–56]. It remains to be seen whether (similar to *C*. *elegans*) KYNA levels fluctuate with nutrient status in mammals and if changes in the KP contribute to learning improvements associated with DR or genetic and pharmacological interventions that mimic aspects of DR.

We found that a short-term fast was as effective at enhancing learning as chronic DR mimetics such as decreased insulin or mTOR signaling. In mammals, long-term exposure to DR activates additional mechanisms, such as increased brain-derived neurotrophic factor (BDNF) expression and enhanced neurogenesis, that also contribute to learning [57,58]. Because the levels of KYNA are exquisitely sensitive to nutrient status, its role may be in making the nervous system quickly responsive to DR. We speculate that the effects that are sensed by KYNA may then be maintained via other mechanisms involved in DR-mediated plasticity: intermediate-term changes such as translation of synaptic proteins and long-term changes such as dendritic spine remodeling or neurogenesis.

Overall, this work points to KYNA as an evolutionarily conserved inhibitory neuromodulator whose levels directly link the metabolic state of the periphery to neuronal functions involved in learning. As such, they reveal a specific mechanism that underlies the beneficial effects of DR on learning independent of the effects on lifespan.

## Materials and methods

### Strains

Mutant and transgenic strains used in this study were: *nmr-1(ak4)*, *nmr-2(tm3785)*, *glr-4(tm3239)*, *kmo-1(tm4529)*, *haao-1(tm4627)*, *nkat-1(ok566)*, *nkat-1(ok566); kmo-1(tm4529)*, *odr-1(n1936)*, *N2; Ex[Pceh-36*:*ced-3; Podr-1*:*GFP; Pmyo-2*:*mcherry] Is[Pmex-5*:*GFP*:*his58*:*odr1*.*7*:*tbb2]*, *eat-2(ad465)*, *tph-1(mg280)*, *flp-18(gk3063)*, *tdc-1(ok914)*, *tbh-1(ok1196)*, *dbl-1(nk3)*, *daf-2(e1370)*, *nmr-1(ak4); Ex[nmr-1p*::*nmr-1*::*bcsGFP; unc-122p*::*GFP]*, *nmr-1(ak4); Ex[cex-1p*::*nmr-1*::*bcsGFP; unc-122p*::*GFP]*, *nmr-1(ak4); Ex[rig-3p*::*nmr-1*::*bcsGFP; odr-1p*::*CHERRY]*, *N2; Ex[cex-1p*::*pkc-1(gf)*::*bcsGFP; odr-1p*::*CHERRY]*, *N2; Ex[cex-1p*::*unc-103(gf)*::*bcsCHERRY; unc-122p*::*GFP]*, *nkat-1(ok566); Ex[nkat-1p*::*nkat-1*::*bcsGFP; unc-122p*::*GFP]*, *nkat-1(ok566); Ex[cex-1p*::*nkat-1*::*bcsGFP; unc-122p*::*GFP]*, *nkat-1(ok566); Ex[exp-1*::*nkat-1*::*bcsCHERRY; unc-122p*::*GFP]*, *N2; Ex[cex-1p*::*GCaMP3*.*0; tdc-1p*::*CHERRY]*, *nmr-1(ak4); Ex[cex-1p*::*GCaMP3*.*0; tdc-1p*::*CHERRY]*, *kmo-1(tm4529); Ex[cex-1p*::*GCaMP3*.*0; tdc-1p*::*mCherry]*, *N2; Ex[nkat-1p*::*GFP; kmo-1p*::*CHERRY]*, *N2; Ex[nkat-1p*::*GFP; tdo-2p*::*CHERRY]*, *daf-16(mu86)*, *skn-1(EU1)*, and *hlh-30(tm1978)*. Transgenic lines were prepared via microinjection of DNA plasmids. Unless described otherwise, *C*. *elegans* were cultured on agar plates with OP50 *E*. *coli* or HT-115 *E*. *coli* encoding relevant double-stranded RNAi.

### Plasmids

Plasmids were constructed using standard techniques. *cex-1p* was used as an RIM-specific promoter. *exp-1p* was used to express genes in RID, and *rig-3p* was used for AVA expression.

### Materials

Chemicals were purchased from Sigma. HT-115 *E*. *coli* encoding double-stranded RNAi were from existing libraries [59,60].

### Short-term butanone associative learning assay

Short-term associative learning was performed as previously described [11]. Animals were grown, conditioned, and assayed at 20°C. When RNAi was used, animals were grown on vector RNAi until the L4 stage and transferred to the appropriate RNAi bacteria until adulthood; animals were conditioned on the corresponding RNAi bacteria.

### Diacetyl learning assay

Diacetyl learning was performed as previously described [25]. Animals were grown, conditioned, and assayed at 20°C.

### NaCl learning assay

NaCl learning was performed as previously described [26]. Animals were grown on plates with 50 mM NaCl and conditioned on plates with 150 mM NaCl with or without food for 3 hours or 6 hours. Animals were grown, conditioned, and assayed at 20°C.

### Culture of *C*. *elegans* for metabolite determination

Synchronized wild-type L1 animals were grown in 15-ml liquid cultures of 20,000 animals in S-Medium and vector RNAi bacteria at an OD of 9 and allowed to shake at 150 RPM at 20°C. At the L3 stage, animals were collected, washed, and placed in new cultures with corresponding RNAi bacteria. (*daf-2(e1370)* mutants were cultured on OP50 bacteria at 15°C until L3 and then shifted to 20°C to prevent them from entering the dauer stage.) At the L4 stage, animals were collected, placed at 4°C, washed 3 times in S-basal, washed once in a 10 mM pH 6.0 potassium citrate buffer, frozen in liquid nitrogen, and placed at −80°C until metabolite extraction.

### Metabolite extraction

Metabolite extraction and determination were performed as previously described [18]. Samples were thawed on ice and 5 g/ml ZrO beads (0.5 mm in diameter) were added. Samples were lysed using a bead beater at 4°C (6 cycles of 20 seconds on followed by 1 minute off). Lysed samples were separated from beads using a 200-μl pipet and then centrifuged. 20 μl 50% w/v trichloroacetic acid in water was added to supernatants to precipitate proteins and incubated at 4°C for 30 minutes. They were then centrifuged at 4°C and the deproteinized supernatants were collected. Extracts were frozen at −80°C until metabolite determination.

### Metabolite determination

Extracts (100 μl per sample) were separated on a Zorbax Eclipse-plus C18 reverse-phase column (4.6 x 150 mm, 100 A pore size, 3.5 μm particle size) equipped with a 4.6 x 10 mM C18 guard column using an isocratic elution of a mobile phase containing 250 mM zinc acetate/acetic acid pH 6.2 and 4% (v/v) acetonitrile at a flow rate of 1 ml/minute. KYNA was detected with an inline fluorescence detector set at 350 nM excitation and 415 nM emission wavelengths. The concentration of KYNA in the extracts was determined by comparison of peak areas to that of standard samples of known concentration prepared in 5% trichloroacetic acid, eluted and detected under identical conditions.

### Spontaneous Ca^2+^ transients

Day 1 adult *Ex[cex-1p*::*GCaMP3*.*0; tdc-1p*::*CHERRY]* animals were conditioned as in the short-term butanone associative learning assay, mounted on agarose pads, and immobilized with cover slips. They were imaged for 250 seconds using a 60x objective on a microscope with green epifluorescence acquiring 4x4 binned images at 2 Hz. A square 6x6 pixel region of interest was drawn around RIM, and integrated fluorescence intensity was measured in each frame. An average baseline intensity was calculated, and any values that were at least 30% more intense than baseline were scored as Ca^2+^ transients. The average total integrated intensity for each imaging period was then calculated.

### Real-time qPCR

RNA extraction, cDNA preparation, and real-time qPCR were performed as previously described [61] from 3 biological replicates of each condition. Data were standardized to actin (*act-1*), and primer sequences are listed in S1 Table.

### Lifespan assay

Lifespan assays were performed at 20°C according to standard protocols. Animals were grown on vector RNAi until the L4 stage and then transferred to the appropriate RNAi. Assays began at day 1 of adulthood with no more than 30 animals per plate. Every second day, animals were poked 3 times with a platinum wire. Those that did not move were scored as dead. Those that did move were scored as alive and moved to a new plate. Animals that could not be found were scored as censored.

### Pharyngeal pumping

The number of contractions of the posterior pharyngeal bulb of L4 stage animals grown at 20°C was counted for 10 seconds as previously described [62]. To assay post-fast pharyngeal pumping, L4 animals were fasted on NGM plates for 2 hours and then contractions of the pharyngeal bulb were counted 5 minutes after they were returned to NGM plates with food.

### Confocal microscopy

*C*. *elegans* were immobilized on 2% agarose pads containing NaN_3_ and imaged under a cover slip through a 60X (1.0 NA) water immersion objective using a Nikon C1si point-scanning confocal microscope equipped with 488-nm and 561-nm lasers as excitation sources for GFP and mCherry, respectively. Images presented are the average-intensity projections of 5 serial, 1-μm-spaced Z-sections.

### Statistical analyses

On all bar graphs, bars represent mean + SEM. Two-tailed Student *t* tests were used for comparisons between 2 conditions. One- or two-way ANOVAs were used for comparisons between multiples conditions, and appropriate post-tests were used. When all conditions were compared to 1 control, a Tukey correction was used. When conditions were compared among themselves, a Bonferroni correction was used. Logrank tests were used for comparisons of survival curves.

## Supporting information

S1 Fig Short term associative learning assay, related to Fig 1. Ad libitum fed animals at day 1 of adulthood were put through one of three conditioning manipulations before being assessed in a chemotaxis assay.
(TIF)

S2 Fig Requirements of NMDAR and RIM function, related to Fig 1. (A) Reconstitution of nmr-1 in AVA rescues its post-fast feeding phenotype. Pharyngeal pumping rate was measured when fasted animals were reintroduced to food. Values are represented as percentage of ad libitum fed pumping rate. tg denotes transgenic animals; non-tg denotes non-transgenic siblings. n = 10, *p<0.05, ***p<0.001 by one-way ANOVA (Tukey). (B) Average intensity of nmr-1 mutants’ spontaneous GCaMP transients in RIM from the 250s imaging window aligned to a -5s to 20s time axis. n = 6–10. (C) Average total intensity of RIM GCaMP flouresence in nmr-1 mutants over the entire 250s imaging window shows no significant change when fasted and conditioned. n = 6–10, by one-way ANOVA (Tukey). (D) Chronic activation or inactivation of RIM has no effect on naïve chemotaxis to butanone. n = 3–6, by one-way ANOVA (Tukey). All data are represented as mean ± SEM. Underlying data can be found in S1 Data.
(TIF)

S3 Fig The beneficial effects of KYNA depletion on learning require NMDARs, related to Fig 2. (A) Learning index values for animals that have been fasted for one hour and then conditioned for one hour. n = 6–10, ***p<0.001 by one-way ANOVA (Bonferroni). (B) Learning index values for animals that have been fasted for one hour and then conditioned for 4 hours. n = 6, ***p<0.001 by one-way ANOVA (Tukey). (C) Learning index values for kmo-1 mutants given chronic DR (50–90% of ad lib. food levels for their entire lives). n = 3, *p<0.05 bye one-way ANOVA (Tukey). (D) Learning index values for nkat-1 mutants given chronic DR (50–90% of ad lib. food levels for their entire lives). n = 3, significance measured by one-way ANOVA (Tukey). (E) Reconstitution of nkat-1 in the RID interneuron is sufficient to restore nkat-1 mutants’ hyperactivated feeding to wild-type levels. tg denotes transgenic animals; non-tg denotes non-transgenic siblings. n = 10, **p<0.01 by one-way ANOVA (Tukey). (F) Average intensity of spontaneous GCaMP transients in RIM of animals on nkat-1 RNAi from the 250s imaging window aligned to a -5s to 20s time axis. n = 6–10. (G) Average intensity of kmo-1 mutants’ spontaneous GCaMP transients in RIM from the 250s imaging window aligned to a -5s to 20s time axis. n = 6–10. All data are represented as mean ± SEM. Underlying data can be found in S1 Data.
(TIF)

S4 Fig Fasting enhances NaCl aversion learning, related to Fig 3. NaCl aversion short term training: learning index values for animals fasted in normal NaCl for 3 hours and subsequently conditioned with high NaCl without food for 3 hours. n = 3, *p<0.05 by one-way ANOVA (Bonferroni). All data are represented as mean ± SEM. Underlying data can be found in S1 Data.
(TIF)

S5 Fig GCaMP traces of animals given DR mimetics, related to Fig 4. (A-F) Average intensity of spontaneous GCaMP transients in RIM of animals on DR mimetics from the 250s imaging window aligned to a -5s to 20s time axis. n = 6–10. Underlying data can be found in S1 Data.
(TIF)

S6 Fig KP modulation does not affect lifespan, related to Table 1. (A-G) Survival curves for wild-type, kmo-1, and nkat-1 animals given DR mimetics. Significance measured by logrank test. Median lifespans and n can be found in Table 1.
(TIF)

S7 Fig KP modulation does not affect lifespan, related to Table 1. (A-G) Survival curves for second replicates of wild-type, kmo-1, and nkat-1 animals given DR mimetics. Significance measured by logrank test. Median lifespans and n can be found in Table 1.
(TIF)

S1 Table Primer sequences used for real-time qPCR, related to Fig 5. (XLSX)

S1 Data Numerical data used for Figs 1A–1J, 2B–2G, 3A–3D, 4A–4H and 5D–5G and S2, S3, S4 and S5 Figs. (XLSX)

## Abbreviations

AMPA: α-amino-3-hydroxy-5-methyl-4-isoxazolepropionic acid
AMPAR: AMPA receptor
AMPK: AMP-activated protein kinase
BDNF: brain-derived neurotrophic factor
DR: dietary restriction
GFP: green fluorescent protein
HPLC: high-performance liquid chromatography
KP: kynurenine pathway
KYNA: kynurenic acid
mTOR: mechanistic target of rapamycin
NMDAR: N-methyl D-aspartate receptor
PKC: protein kinase C
QA: quinolinic acid
qPCR: quantitative PCR
RNAi: RNA interference
TGFβ: transforming growth factor β

## References

[1] Murphy T, Dias GP, Thuret S. Effects of diet on brain plasticity in animal and human studies: mind the gap. Neural Plast. 2014;2014: 563160 doi: 10.1155/2014/563160 24900924 10.1155/2014/563160 PMC4037119

[2] Kenyon CJ. The genetics of ageing. Nature. 2010;464: 504–512. doi: 10.1038/nature08980 20336132 10.1038/nature08980

[3] López-Otín C, Blasco MA, Partridge L, Serrano M, Kroemer G. The hallmarks of aging. Cell. 2013;153: 1194–1217. doi: 10.1016/j.cell.2013.05.039 23746838 10.1016/j.cell.2013.05.039 PMC3836174

[4] Stanley M, Macauley SL, Holtzman DM. Changes in insulin and insulin signaling in Alzheimer’s disease: cause or consequence? J Exp Med. 2016;213: 1375–1385. doi: 10.1084/jem.20160493 27432942 10.1084/jem.20160493 PMC4986537

[5] Dal-Pan A, Pifferi F, Marchal J, Picq J-L, Aujard F, RESTRIKAL Consortium. Cognitive performances are selectively enhanced during chronic caloric restriction or resveratrol supplementation in a primate. PLoS ONE. 2011;6: e16581 doi: 10.1371/journal.pone.0016581 21304942 10.1371/journal.pone.0016581 PMC3031601

[6] Riddle MC, McKenna MC, Yoon YJ, Pattwell SS, Santos PMG, Casey BJ, et al Caloric restriction enhances fear extinction learning in mice. Neuropsychopharmacol Off Publ Am Coll Neuropsychopharmacol. 2013;38: 930–937. doi: 10.1038/npp.2012.268 23303073 10.1038/npp.2012.268 PMC3629393

[7] Maalouf M, Rho JM, Mattson MP. The neuroprotective properties of calorie restriction, the ketogenic diet, and ketone bodies. Brain Res Rev. 2009;59: 293–315. doi: 10.1016/j.brainresrev.2008.09.002 18845187 10.1016/j.brainresrev.2008.09.002 PMC2649682

[8] Greer EL, Brunet A. Different dietary restriction regimens extend lifespan by both independent and overlapping genetic pathways in C. elegans. Aging Cell. 2009;8: 113–127. doi: 10.1111/j.1474-9726.2009.00459.x 19239417 10.1111/j.1474-9726.2009.00459.x PMC2680339

[9] Colbert HA, Bargmann CI. Odorant-specific adaptation pathways generate olfactory plasticity in C. elegans. Neuron. 1995;14: 803–812. 7718242 10.1016/0896-6273(95)90224-4

[10] Kano T, Brockie PJ, Sassa T, Fujimoto H, Kawahara Y, Iino Y, et al Memory in Caenorhabditis elegans is mediated by NMDA-type ionotropic glutamate receptors. Curr Biol CB. 2008;18: 1010–1015. doi: 10.1016/j.cub.2008.05.051 18583134 10.1016/j.cub.2008.05.051 PMC2645413

[11] Kauffman AL, Ashraf JM, Corces-Zimmerman MR, Landis JN, Murphy CT. Insulin signaling and dietary restriction differentially influence the decline of learning and memory with age. PLoS Biol. 2010;8: e1000372 doi: 10.1371/journal.pbio.1000372 20502519 10.1371/journal.pbio.1000372 PMC2872642

[12] Torayama I, Ishihara T, Katsura I. Caenorhabditis elegans integrates the signals of butanone and food to enhance chemotaxis to butanone. J Neurosci Off J Soc Neurosci. 2007;27: 741–750. doi: 10.1523/JNEUROSCI.4312-06.2007 17251413 10.1523/JNEUROSCI.4312-06.2007 PMC6672901

[13] Morris RG, Anderson E, Lynch GS, Baudry M. Selective impairment of learning and blockade of long-term potentiation by an N-methyl-D-aspartate receptor antagonist, AP5. Nature. 1986;319: 774–776. doi: 10.1038/319774a0 2869411 10.1038/319774a0

[14] Kessler M, Terramani T, Lynch G, Baudry M. A glycine site associated with N-methyl-D-aspartic acid receptors: characterization and identification of a new class of antagonists. J Neurochem. 1989;52: 1319–1328. 2538568 10.1111/j.1471-4159.1989.tb01881.x

[15] Bargmann CI, Hartwieg E, Horvitz HR. Odorant-selective genes and neurons mediate olfaction in C. elegans. Cell. 1993;74: 515–527. 8348618 10.1016/0092-8674(93)80053-h

[16] Sprengel R, Aronoff R, Völkner M, Schmitt B, Mosbach R, Kuner T. Glutamate receptor channel signatures. Trends Pharmacol Sci. 2001;22: 7–10. 11165660 10.1016/s0165-6147(00)01588-1

[17] Brockie PJ, Madsen DM, Zheng Y, Mellem J, Maricq AV. Differential expression of glutamate receptor subunits in the nervous system of Caenorhabditis elegans and their regulation by the homeodomain protein UNC-42. J Neurosci Off J Soc Neurosci. 2001;21: 1510–1522. 10.1523/JNEUROSCI.21-05-01510.2001 PMC6762961 11222641

[18] Lemieux GA, Cunningham KA, Lin L, Mayer F, Werb Z, Ashrafi K. Kynurenic acid is a nutritional cue that enables behavioral plasticity. Cell. 2015;160: 119–131. doi: 10.1016/j.cell.2014.12.028 25594177 10.1016/j.cell.2014.12.028 PMC4334586

[19] Gordus A, Pokala N, Levy S, Flavell SW, Bargmann CI. Feedback from network states generates variability in a probabilistic olfactory circuit. Cell. 2015;161: 215–227. doi: 10.1016/j.cell.2015.02.018 25772698 10.1016/j.cell.2015.02.018 PMC4821011

[20] Brockie PJ, Mellem JE, Hills T, Madsen DM, Maricq AV. The C. elegans glutamate receptor subunit NMR-1 is required for slow NMDA-activated currents that regulate reversal frequency during locomotion. Neuron. 2001;31: 617–630. 11545720 10.1016/s0896-6273(01)00394-4

[21] Macosko EZ, Pokala N, Feinberg EH, Chalasani SH, Butcher RA, Clardy J, et al A hub-and-spoke circuit drives pheromone attraction and social behaviour in C. elegans. Nature. 2009;458: 1171–1175. doi: 10.1038/nature07886 19349961 10.1038/nature07886 PMC2760495

[22] Cho CE, Brueggemann C, L’Etoile ND, Bargmann CI. Parallel encoding of sensory history and behavioral preference during Caenorhabditis elegans olfactory learning. eLife. 2016;5 doi: 10.7554/eLife.14000 27383131 10.7554/eLife.14000 PMC4935464

[23] Stone TW, Perkins MN. Quinolinic acid: a potent endogenous excitant at amino acid receptors in CNS. Eur J Pharmacol. 1981;72: 411–412. 6268428 10.1016/0014-2999(81)90587-2

[24] van der Goot AT, Zhu W, Vázquez-Manrique RP, Seinstra RI, Dettmer K, Michels H, et al Delaying aging and the aging-associated decline in protein homeostasis by inhibition of tryptophan degradation. Proc Natl Acad Sci U S A. 2012;109: 14912–14917. doi: 10.1073/pnas.1203083109 22927396 10.1073/pnas.1203083109 PMC3443121

[25] Hadziselimovic N, Vukojevic V, Peter F, Milnik A, Fastenrath M, Fenyves BG, et al Forgetting is regulated via Musashi-mediated translational control of the Arp2/3 complex. Cell. 2014;156: 1153–1166. doi: 10.1016/j.cell.2014.01.054 24630719 10.1016/j.cell.2014.01.054

[26] Kunitomo H, Sato H, Iwata R, Satoh Y, Ohno H, Yamada K, et al Concentration memory-dependent synaptic plasticity of a taste circuit regulates salt concentration chemotaxis in Caenorhabditis elegans. Nat Commun. 2013;4: 2210 doi: 10.1038/ncomms3210 23887678 10.1038/ncomms3210

[27] Murakami H, Bessinger K, Hellmann J, Murakami S. Aging-dependent and -independent modulation of associative learning behavior by insulin/insulin-like growth factor-1 signal in Caenorhabditis elegans. J Neurosci Off J Soc Neurosci. 2005;25: 10894–10904. doi: 10.1523/JNEUROSCI.3600-04.2005 16306402 10.1523/JNEUROSCI.3600-04.2005 PMC6725869

[28] Jones KT, Greer ER, Pearce D, Ashrafi K. Rictor/TORC2 regulates Caenorhabditis elegans fat storage, body size, and development through sgk-1. PLoS Biol. 2009;7: e60 doi: 10.1371/journal.pbio.1000060 19260765 10.1371/journal.pbio.1000060 PMC2650726

[29] Halloran J, Hussong SA, Burbank R, Podlutskaya N, Fischer KE, Sloane LB, et al Chronic inhibition of mammalian target of rapamycin by rapamycin modulates cognitive and non-cognitive components of behavior throughout lifespan in mice. Neuroscience. 2012;223: 102–113. doi: 10.1016/j.neuroscience.2012.06.054 22750207 10.1016/j.neuroscience.2012.06.054 PMC3454865

[30] Majumder S, Caccamo A, Medina DX, Benavides AD, Javors MA, Kraig E, et al Lifelong rapamycin administration ameliorates age-dependent cognitive deficits by reducing IL-1β and enhancing NMDA signaling. Aging Cell. 2012;11: 326–335. doi: 10.1111/j.1474-9726.2011.00791.x 22212527 10.1111/j.1474-9726.2011.00791.x PMC3306461

[31] O’Rourke EJ, Ruvkun G. MXL-3 and HLH-30 transcriptionally link lipolysis and autophagy to nutrient availability. Nat Cell Biol. 2013;15: 668–676. doi: 10.1038/ncb2741 23604316 10.1038/ncb2741 PMC3723461

[32] Beale EG. 5’-AMP-activated protein kinase signaling in Caenorhabditis elegans. Exp Biol Med Maywood NJ. 2008;233: 12–20. 18156301 10.3181/0705-MR-117

[33] Ren P, Lim CS, Johnsen R, Albert PS, Pilgrim D, Riddle DL. Control of C. elegans larval development by neuronal expression of a TGF-beta homolog. Science. 1996;274: 1389–1391. 8910282 10.1126/science.274.5291.1389

[34] Horvitz HR, Chalfie M, Trent C, Sulston JE, Evans PD. Serotonin and octopamine in the nematode Caenorhabditis elegans. Science. 1982;216: 1012–1014. 6805073 10.1126/science.6805073

[35] Alkema MJ, Hunter-Ensor M, Ringstad N, Horvitz HR. Tyramine Functions independently of octopamine in the Caenorhabditis elegans nervous system. Neuron. 2005;46: 247–260. doi: 10.1016/j.neuron.2005.02.024 15848803 10.1016/j.neuron.2005.02.024

[36] Suo S, Kimura Y, Van Tol HHM. Starvation induces cAMP response element-binding protein-dependent gene expression through octopamine-Gq signaling in Caenorhabditis elegans. J Neurosci Off J Soc Neurosci. 2006;26: 10082–10090. doi: 10.1523/JNEUROSCI.0819-06.2006 17021164 10.1523/JNEUROSCI.0819-06.2006 PMC6674634

[37] Raizen DM, Lee RY, Avery L. Interacting genes required for pharyngeal excitation by motor neuron MC in Caenorhabditis elegans. Genetics. 1995;141: 1365–1382. 8601480 10.1093/genetics/141.4.1365 PMC1206873

[38] Avery L. The genetics of feeding in Caenorhabditis elegans. Genetics. 1993;133: 897–917. 8462849 10.1093/genetics/133.4.897 PMC1205408

[39] Lakowski B, Hekimi S. The genetics of caloric restriction in Caenorhabditis elegans. Proc Natl Acad Sci U S A. 1998;95: 13091–13096. 9789046 10.1073/pnas.95.22.13091 PMC23719

[40] Lapierre LR, De Magalhaes Filho CD, McQuary PR, Chu C-C, Visvikis O, Chang JT, et al The TFEB orthologue HLH-30 regulates autophagy and modulates longevity in Caenorhabditis elegans. Nat Commun. 2013;4: 2267 doi: 10.1038/ncomms3267 23925298 10.1038/ncomms3267 PMC3866206

[41] Carpenedo R, Pittaluga A, Cozzi A, Attucci S, Galli A, Raiteri M, et al Presynaptic kynurenate-sensitive receptors inhibit glutamate release. Eur J Neurosci. 2001;13: 2141–2147. 11422455 10.1046/j.0953-816x.2001.01592.x

[42] Hilmas C, Pereira EF, Alkondon M, Rassoulpour A, Schwarcz R, Albuquerque EX. The brain metabolite kynurenic acid inhibits alpha7 nicotinic receptor activity and increases non-alpha7 nicotinic receptor expression: physiopathological implications. J Neurosci Off J Soc Neurosci. 2001;21: 7463–7473. 10.1523/JNEUROSCI.21-19-07463.2001 PMC6762893 11567036

[43] Agudelo LZ, Femenía T, Orhan F, Porsmyr-Palmertz M, Goiny M, Martinez-Redondo V, et al Skeletal muscle PGC-1α1 modulates kynurenine metabolism and mediates resilience to stress-induced depression. Cell. 2014;159: 33–45. doi: 10.1016/j.cell.2014.07.051 25259918 10.1016/j.cell.2014.07.051

[44] Schwarcz R, Bruno JP, Muchowski PJ, Wu H-Q. Kynurenines in the mammalian brain: when physiology meets pathology. Nat Rev Neurosci. 2012;13: 465–477. doi: 10.1038/nrn3257 22678511 10.1038/nrn3257 PMC3681811

[45] Potter MC, Elmer GI, Bergeron R, Albuquerque EX, Guidetti P, Wu H-Q, et al Reduction of endogenous kynurenic acid formation enhances extracellular glutamate, hippocampal plasticity, and cognitive behavior. Neuropsychopharmacol Off Publ Am Coll Neuropsychopharmacol. 2010;35: 1734–1742. doi: 10.1038/npp.2010.39 20336058 10.1038/npp.2010.39 PMC3055476

[46] Pocivavsek A, Wu H-Q, Potter MC, Elmer GI, Pellicciari R, Schwarcz R. Fluctuations in endogenous kynurenic acid control hippocampal glutamate and memory. Neuropsychopharmacol Off Publ Am Coll Neuropsychopharmacol. 2011;36: 2357–2367. doi: 10.1038/npp.2011.127 21796108 10.1038/npp.2011.127 PMC3176574

[47] Bonda DJ, Mailankot M, Stone JG, Garrett MR, Staniszewska M, Castellani RJ, et al Indoleamine 2,3-dioxygenase and 3-hydroxykynurenine modifications are found in the neuropathology of Alzheimer’s disease. Redox Rep Commun Free Radic Res. 2010;15: 161–168. doi: 10.1179/174329210X12650506623645 20663292 10.1179/174329210X12650506623645 PMC2956440

[48] Guillemin GJ, Brew BJ, Noonan CE, Takikawa O, Cullen KM. Indoleamine 2,3 dioxygenase and quinolinic acid immunoreactivity in Alzheimer’s disease hippocampus. Neuropathol Appl Neurobiol. 2005;31: 395–404. doi: 10.1111/j.1365-2990.2005.00655.x 16008823 10.1111/j.1365-2990.2005.00655.x

[49] Beal MF, Matson WR, Storey E, Milbury P, Ryan EA, Ogawa T, et al Kynurenic acid concentrations are reduced in Huntington’s disease cerebral cortex. J Neurol Sci. 1992;108: 80–87. 1385624 10.1016/0022-510x(92)90191-m

[50] Pearson SJ, Reynolds GP. Increased brain concentrations of a neurotoxin, 3-hydroxykynurenine, in Huntington’s disease. Neurosci Lett. 1992;144: 199–201. 1436703 10.1016/0304-3940(92)90749-w

[51] Ogawa T, Matson WR, Beal MF, Myers RH, Bird ED, Milbury P, et al Kynurenine pathway abnormalities in Parkinson’s disease. Neurology. 1992;42: 1702–1706. 1513457 10.1212/wnl.42.9.1702

[52] Steiner J, Walter M, Gos T, Guillemin GJ, Bernstein H-G, Sarnyai Z, et al Severe depression is associated with increased microglial quinolinic acid in subregions of the anterior cingulate gyrus: evidence for an immune-modulated glutamatergic neurotransmission? J Neuroinflammation. 2011;8: 94 doi: 10.1186/1742-2094-8-94 21831269 10.1186/1742-2094-8-94 PMC3177898

[53] Schwarcz R, Rassoulpour A, Wu HQ, Medoff D, Tamminga CA, Roberts RC. Increased cortical kynurenate content in schizophrenia. Biol Psychiatry. 2001;50: 521–530. 11600105 10.1016/s0006-3223(01)01078-2

[54] Campesan S, Green EW, Breda C, Sathyasaikumar KV, Muchowski PJ, Schwarcz R, et al The kynurenine pathway modulates neurodegeneration in a Drosophila model of Huntington’s disease. Curr Biol CB. 2011;21: 961–966. doi: 10.1016/j.cub.2011.04.028 21636279 10.1016/j.cub.2011.04.028 PMC3929356

[55] Savvateeva E, Popov A, Kamyshev N, Bragina J, Heisenberg M, Senitz D, et al Age-dependent memory loss, synaptic pathology and altered brain plasticity in the Drosophila mutant cardinal accumulating 3-hydroxykynurenine. J Neural Transm Vienna Austria 1996. 2000;107: 581–601. 10.1007/s007020070080 11072753

[56] Zwilling D, Huang S-Y, Sathyasaikumar KV, Notarangelo FM, Guidetti P, Wu H-Q, et al Kynurenine 3-monooxygenase inhibition in blood ameliorates neurodegeneration. Cell. 2011;145: 863–874. doi: 10.1016/j.cell.2011.05.020 21640374 10.1016/j.cell.2011.05.020 PMC3118409

[57] Lee J, Duan W, Long JM, Ingram DK, Mattson MP. Dietary restriction increases the number of newly generated neural cells, and induces BDNF expression, in the dentate gyrus of rats. J Mol Neurosci MN. 2000;15: 99–108. doi: 10.1385/JMN:15:2:99 11220789 10.1385/JMN:15:2:99

[58] Lee J, Seroogy KB, Mattson MP. Dietary restriction enhances neurotrophin expression and neurogenesis in the hippocampus of adult mice. J Neurochem. 2002;80: 539–547. 11905999 10.1046/j.0022-3042.2001.00747.x

[59] Dillin A, Crawford DK, Kenyon C. Timing Requirements for Insulin/IGF-1 Signaling in C. elegans. Science. 2002;298: 830–834. doi: 10.1126/science.1074240 12399591 10.1126/science.1074240

[60] Kamath RS, Fraser AG, Dong Y, Poulin G, Durbin R, Gotta M, et al Systematic functional analysis of the Caenorhabditis elegans genome using RNAi. Nature. 2003;421: 231–237. doi: 10.1038/nature01278 12529635 10.1038/nature01278

[61] Van Gilst MR, Hadjivassiliou H, Jolly A, Yamamoto KR. Nuclear hormone receptor NHR-49 controls fat consumption and fatty acid composition in C. elegans. PLoS Biol. 2005;3: e53 doi: 10.1371/journal.pbio.0030053 15719061 10.1371/journal.pbio.0030053 PMC547972

[62] Srinivasan S, Sadegh L, Elle IC, Christensen AGL, Faergeman NJ, Ashrafi K. Serotonin regulates C. elegans fat and feeding through independent molecular mechanisms. Cell Metab. 2008;7: 533–544. doi: 10.1016/j.cmet.2008.04.012 18522834 10.1016/j.cmet.2008.04.012 PMC2495008

