# Supplementary figures and images for: The beneficial effects of dietary restriction on learning are distinct from its effects on longevity and mediated by depletion of a neuroinhibitory metabolite
Source: PLoS Biol. 2017 Aug 1;15(8):e2002032. doi: 10.1371/journal.pbio.2002032 (PMC5538637; doi:10.1371/journal.pbio.2002032)

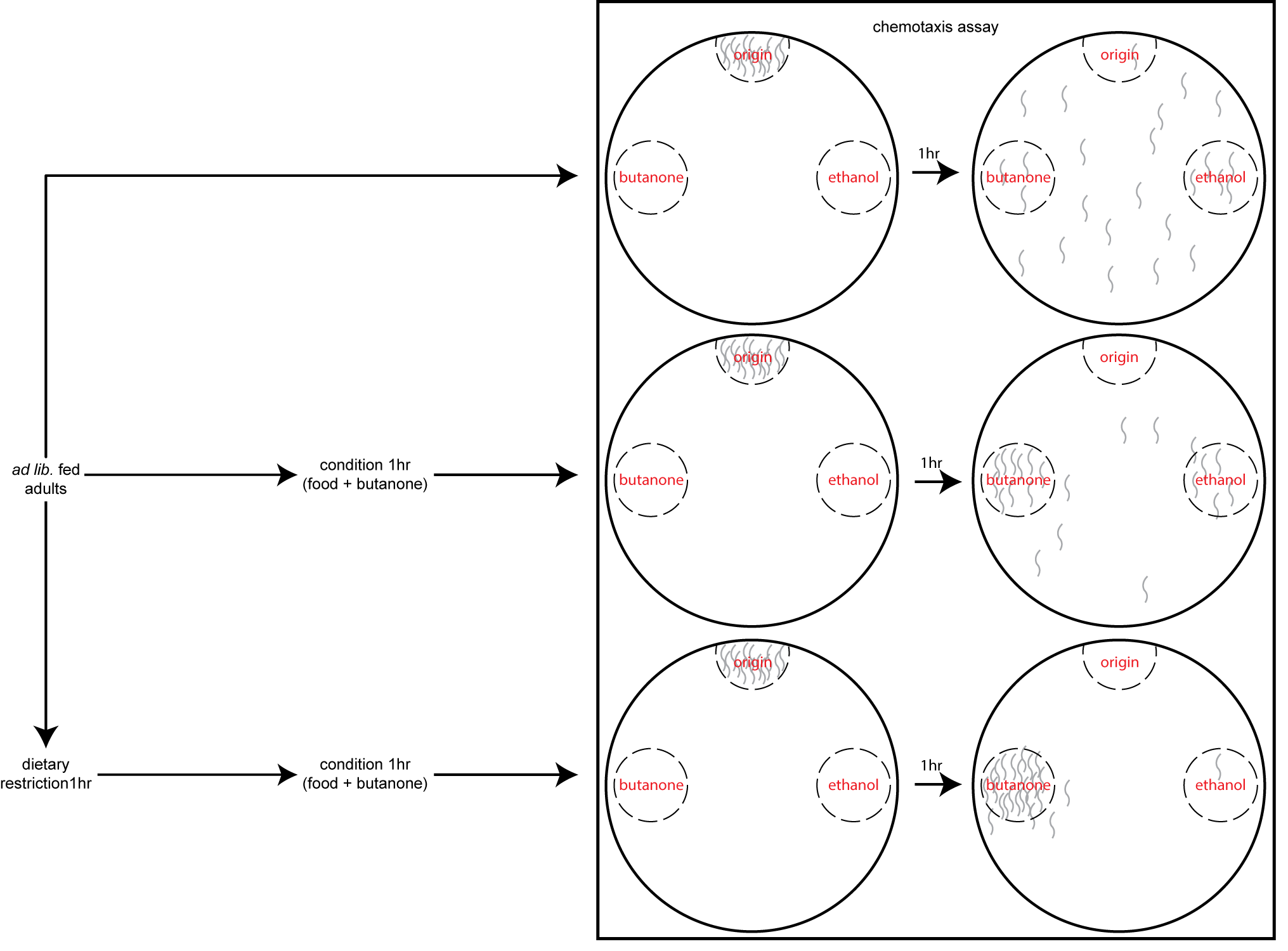

Supplement: S1 Fig — Ad libitum fed animals at day 1 of adulthood were put through one of three conditioning manipulations before being assessed in a chemotaxis assay. (TIF) [file pbio.2002032.s001.tif]

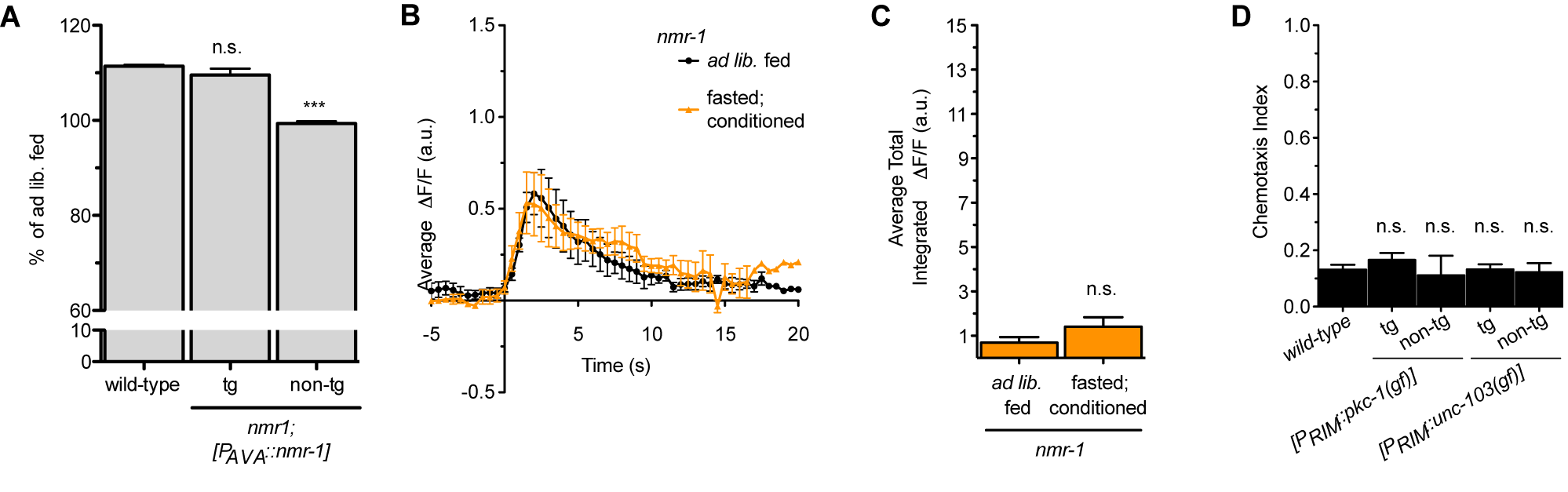

Supplement: S2 Fig — (A) Reconstitution of nmr-1 in AVA rescues its post-fast feeding phenotype. Pharyngeal pumping rate was measured when fasted animals were reintroduced to food. Values are represented as percentage of ad libitum fed pumping rate. tg denotes transgenic animals; non-tg denotes non-transgenic siblings. n = 10, *p<0.05, ***p<0.001 by one-way ANOVA (Tukey). (B) Average intensity of nmr-1 mutants’ spontaneous GCaMP transients in RIM from the 250s imaging window aligned to a -5s to 20s time axis. n = 6–10. (C) Average total intensity of RIM GCaMP flouresence in nmr-1 mutants over the entire 250s imaging window shows no significant change when fasted and conditioned. n = 6–10, by one-way ANOVA (Tukey). (D) Chronic activation or inactivation of RIM has no effect on naïve chemotaxis to butanone. n = 3–6, by one-way ANOVA (Tukey). All data are represented as mean ± SEM. Underlying data can be found in S1 Data. (TIF) [file pbio.2002032.s002.tif]

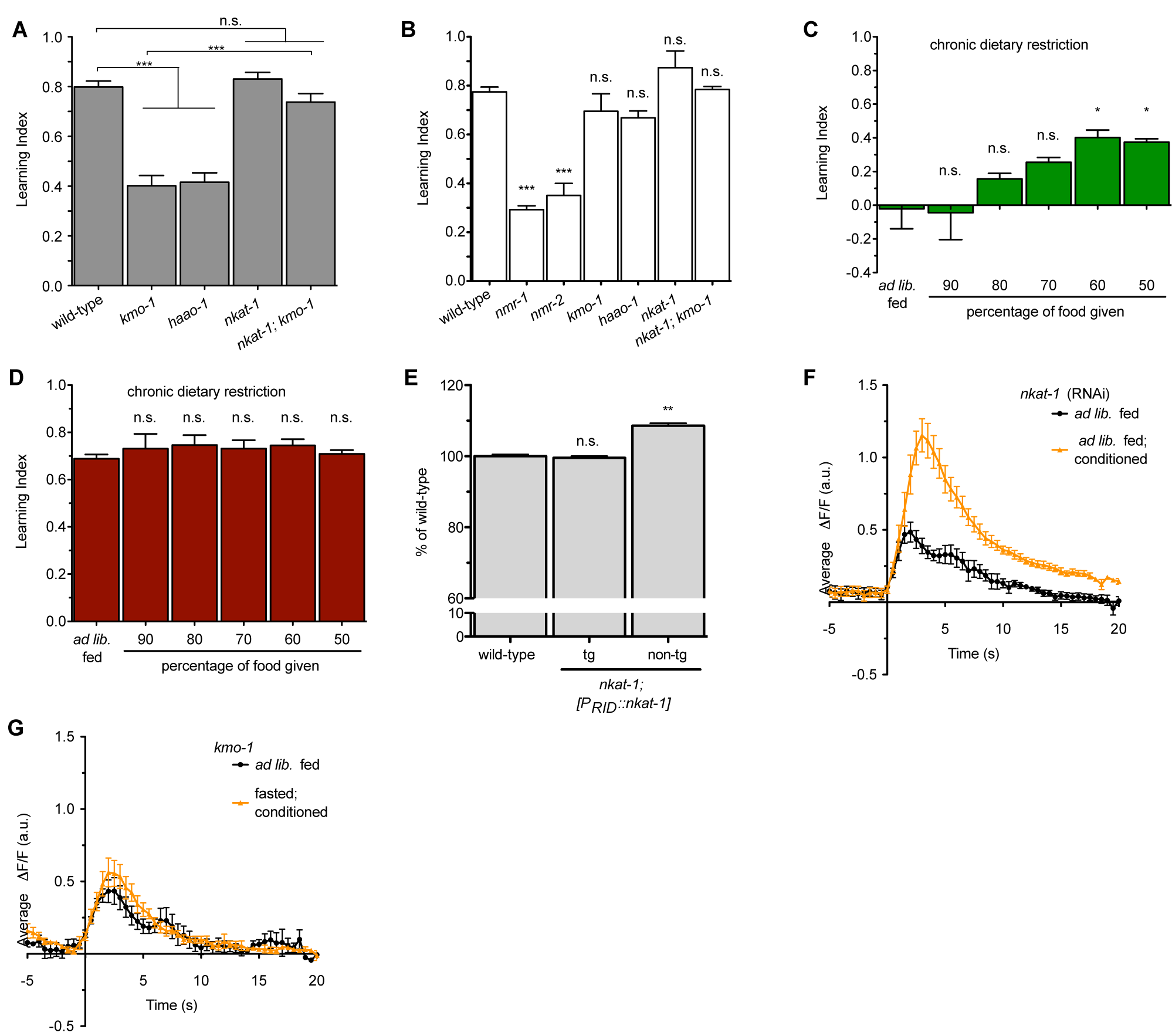

Supplement: S3 Fig — (A) Learning index values for animals that have been fasted for one hour and then conditioned for one hour. n = 6–10, ***p<0.001 by one-way ANOVA (Bonferroni). (B) Learning index values for animals that have been fasted for one hour and then conditioned for 4 hours. n = 6, ***p<0.001 by one-way ANOVA (Tukey). (C) Learning index values for kmo-1 mutants given chronic DR (50–90% of ad lib. food levels for their entire lives). n = 3, *p<0.05 bye one-way ANOVA (Tukey). (D) Learning index values for nkat-1 mutants given chronic DR (50–90% of ad lib. food levels for their entire lives). n = 3, significance measured by one-way ANOVA (Tukey). (E) Reconstitution of nkat-1 in the RID interneuron is sufficient to restore nkat-1 mutants’ hyperactivated feeding to wild-type levels. tg denotes transgenic animals; non-tg denotes non-transgenic siblings. n = 10, **p<0.01 by one-way ANOVA (Tukey). (F) Average intensity of spontaneous GCaMP transients in RIM of animals on nkat-1 RNAi from the 250s imaging window aligned to a -5s to 20s time axis. n = 6–10. (G) Average intensity of kmo-1 mutants’ spontaneous GCaMP transients in RIM from the 250s imaging window aligned to a -5s to 20s time axis. n = 6–10. All data are represented as mean ± SEM. Underlying data can be found in S1 Data. (TIF) [file pbio.2002032.s003.tif]

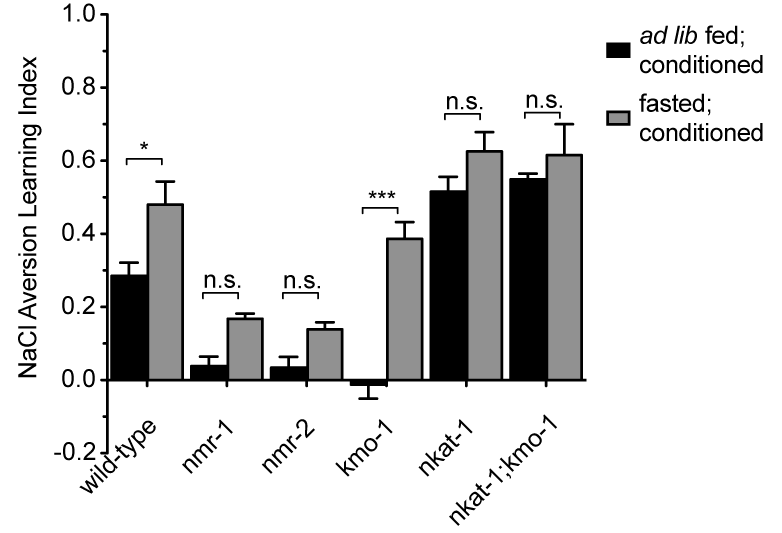

Supplement: S4 Fig — NaCl aversion short term training: learning index values for animals fasted in normal NaCl for 3 hours and subsequently conditioned with high NaCl without food for 3 hours. n = 3, *p<0.05 by one-way ANOVA (Bonferroni). All data are represented as mean ± SEM. Underlying data can be found in S1 Data. (TIF) [file pbio.2002032.s004.tif]

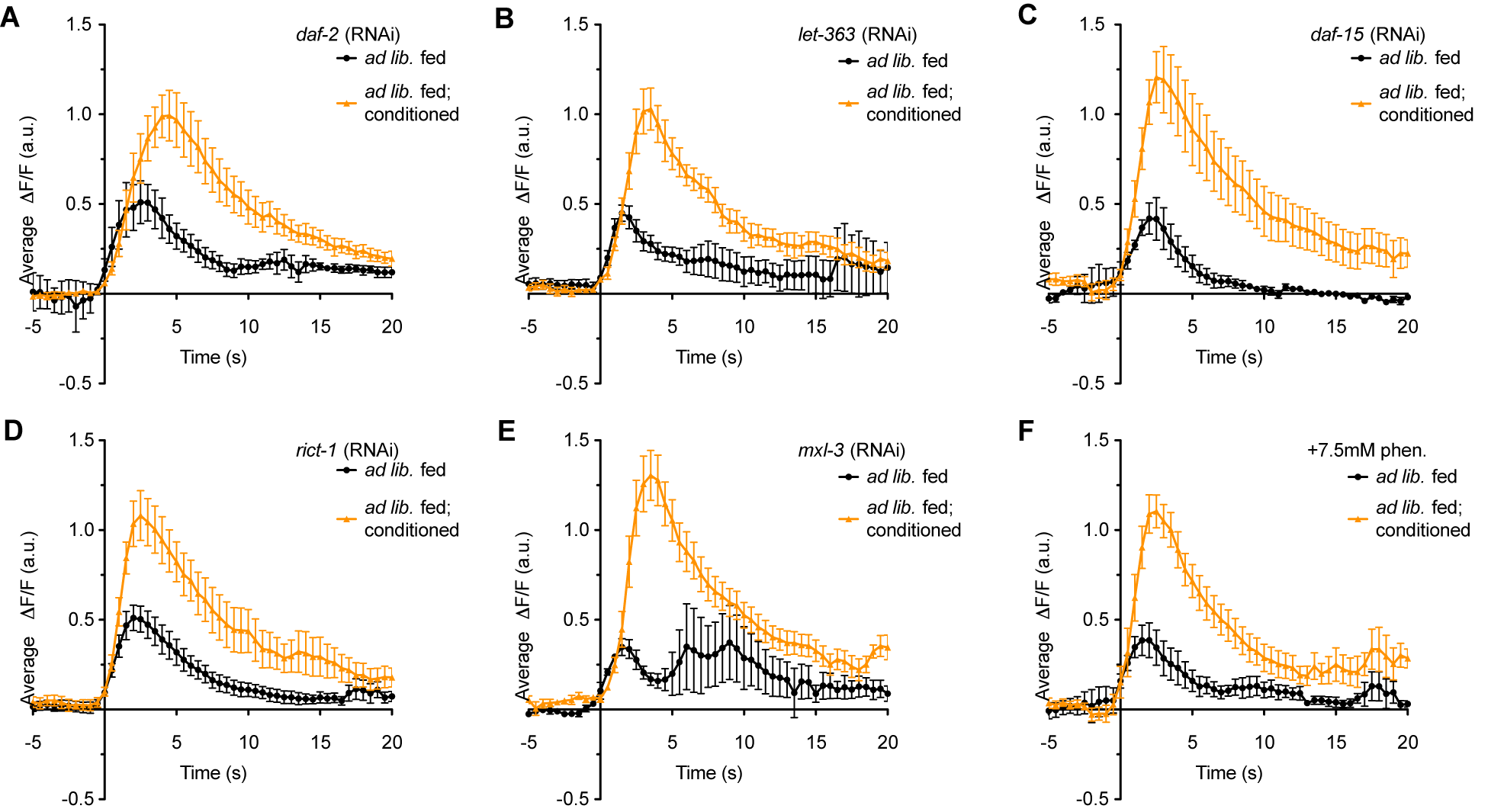

Supplement: S5 Fig — (A-F) Average intensity of spontaneous GCaMP transients in RIM of animals on DR mimetics from the 250s imaging window aligned to a -5s to 20s time axis. n = 6–10. Underlying data can be found in S1 Data. (TIF) [file pbio.2002032.s005.tif]

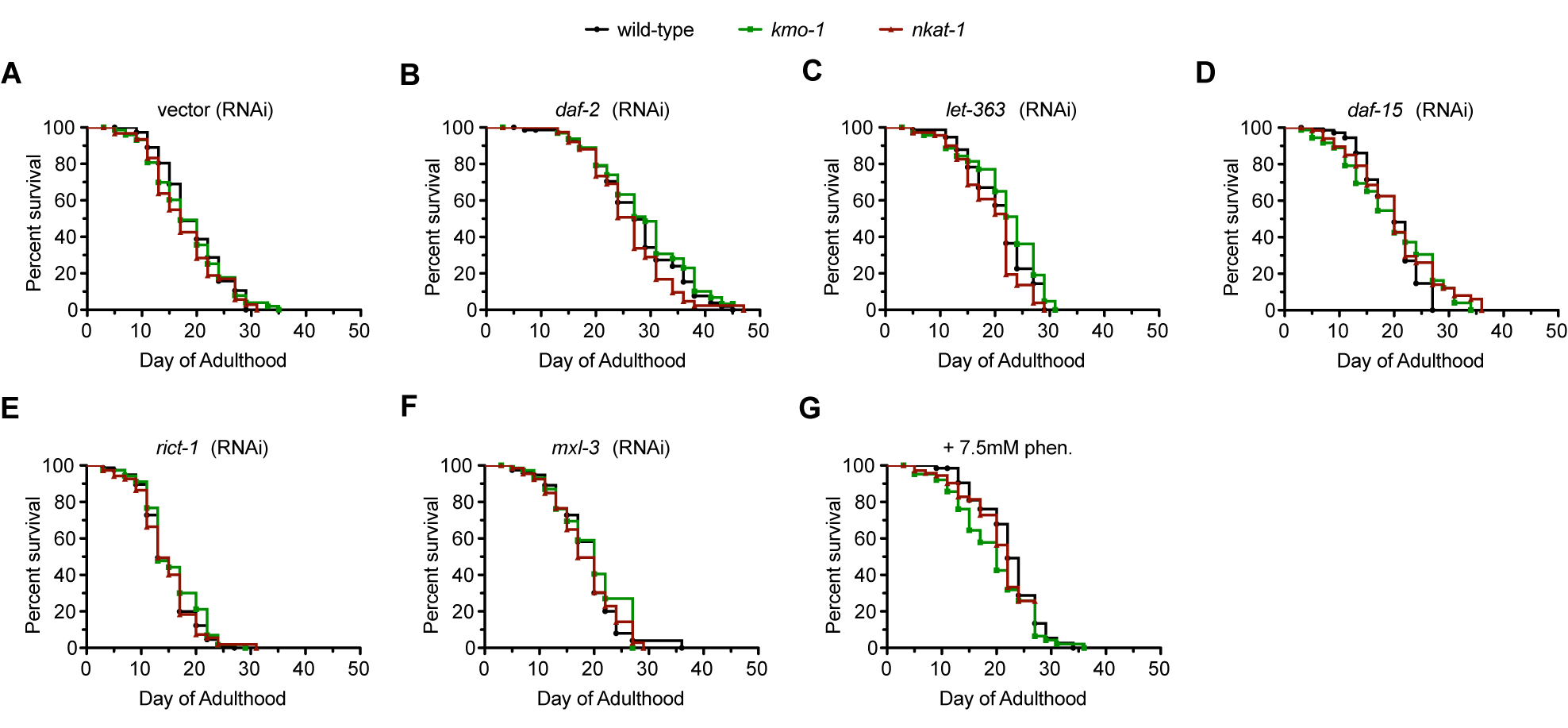

Supplement: S6 Fig — (A-G) Survival curves for wild-type, kmo-1, and nkat-1 animals given DR mimetics. Significance measured by logrank test. Median lifespans and n can be found in Table 1. (TIF) [file pbio.2002032.s006.tif]

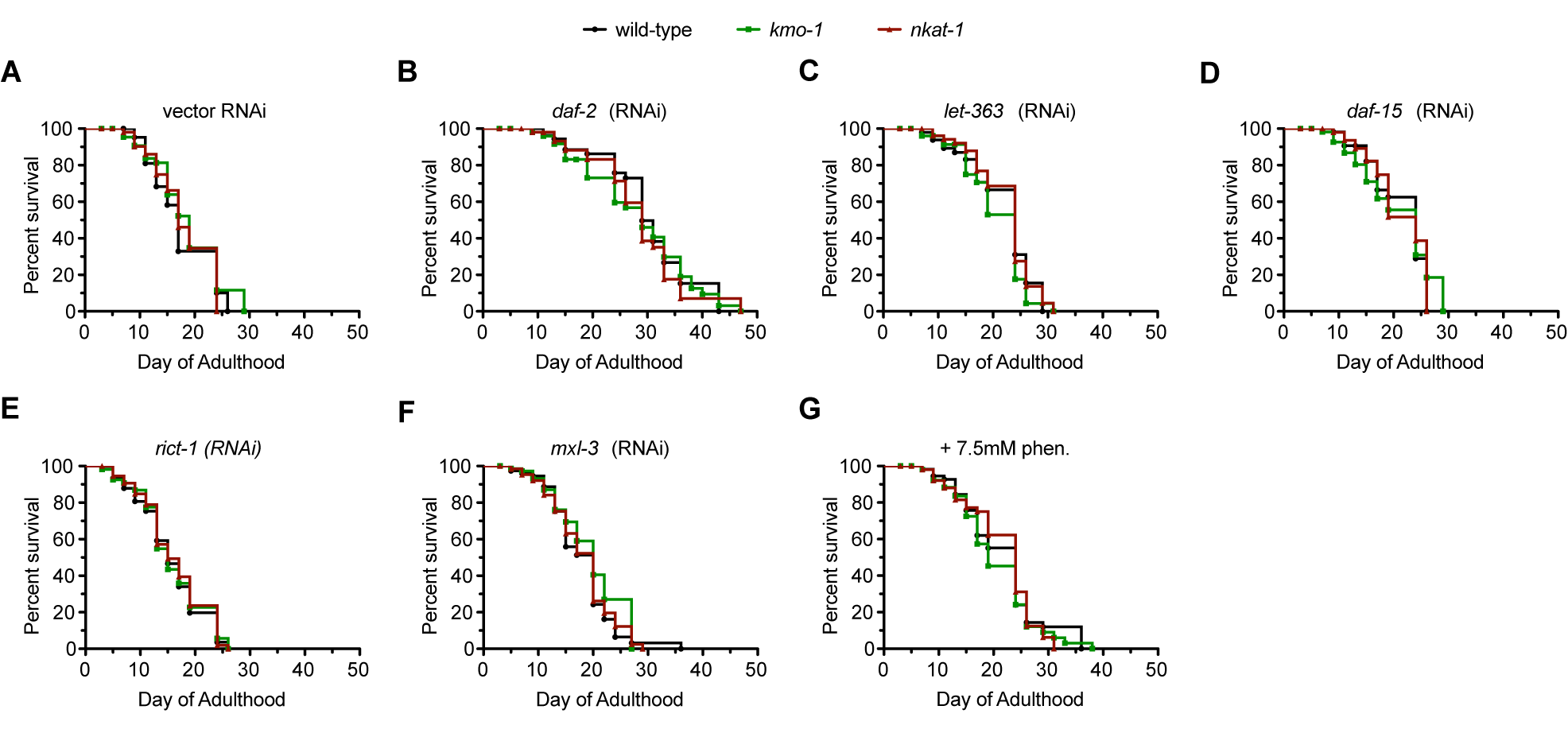

Supplement: S7 Fig — (A-G) Survival curves for second replicates of wild-type, kmo-1, and nkat-1 animals given DR mimetics. Significance measured by logrank test. Median lifespans and n can be found in Table 1. (TIF) [file pbio.2002032.s007.tif]
